# Quantifying rate-limiting genetic variation in breast and ovarian tumourigenesis

**DOI:** 10.1016/j.ebiom.2026.106181

**Published:** 2026-02-21

**Authors:** Kathleen E. Houlahan, Mahad Bihie, Yves Greatti, Julián Grandvallet Contreras, Daniel J. Fulop, Gonzalo Lopez, Marc Williams, Hsin-Hsiung Huang, Peter Van Loo, Paul C. Boutros, Kuan-lin Huang

**Affiliations:** a Department of Human Genetics, University of California, Los Angeles, Los Angeles, CA, USA; b Department of Medical Biophysics, University of Toronto, Toronto, Canada; c Jonsson Comprehensive Cancer Center, David Geffen School of Medicine, University of California, Los Angeles, Los Angeles, CA, USA; d Institute for Precision Health, University of California, Los Angeles, Los Angeles, CA, USA; e Vector Institute, Toronto, Canada; f Department of Medicine, Stanford University School of Medicine, Stanford, CA, USA; g Department of Biochemistry and Biomedical Sciences, McMaster University, Hamilton, ON, Canada; h Centre for Discovery in Cancer Research, McMaster University, Hamilton, ON, Canada; i Johns Hopkins University, Baltimore, USA; j Department of Pediatrics, University of Colorado Anschutz Medical Campus, Aurora, CO, USA; k Department of Genetics and Genomic Sciences, Department of Artificial Intelligence and Human Health, Center for Transformative Disease Modeling, Tisch Cancer Institute, Icahn School of Medicine at Mount Sinai, New York, NY, USA; l Memorial Sloan Kettering Cancer Center, New York, USA; m Department of Statistics, University of Central Florida, Orlando, FL, USA; n The Francis Crick Institute, London, UK; o Department of Genetics, The University of Texas MD Anderson Cancer Center, Houston, TX, USA; p Department of Pharmacology and Toxicology, University of Toronto, Toronto, Canada; q Department of Urology, University of California, Los Angeles, Los Angeles, CA, USA; r Sanford Burnham Prebys Medical Discovery Institute, La Jolla, CA, USA

**Keywords:** Breast cancer, Ovarian cancer, BRCA1, BRCA2, Tumourigenesis, Germline predisposition

## Abstract

**Background:**

The number and type of genetic alterations required to initiate breast and ovarian cancer remain unclear. While germline *BRCA1/2* carriers show markedly elevated cancer risk, it is uncertain whether point mutations or copy number alterations constitute the rate-limiting events of tumourigenesis.

**Methods:**

We developed a statistical framework extending prior incidence–mutation models to estimate the minimal number and type of driver events required for cancer initiation. Somatic mutation and copy-number data from >3000 breast and ovarian cancers in TCGA and METABRIC were compared between germline *BRCA1/2* carriers and non-carriers matched on subtypes. Results were validated through analyses of evolutionary timing data, as well as single-cell whole genome sequencing (scWGS) data of genetically-engineered and patient-derived cancer/pre-cancerous cells.

**Findings:**

Deletions, rather than single-nucleotide variants (SNVs), emerged as the likely rate-limiting events. Modeling indicated that 1–3 deletions are sufficient to initiate tumourigenesis, whereas SNVs alone could not explain observed incidence ratios. *BRCA1/2*-driven and sporadic tumours converged on similar deletion profiles, including early recurrent deletions of chromosomes 13q and 17, though carriers accumulated them more rapidly.

**Interpretation:**

Deletion-associated chromosomal instability likely represents the central trigger for breast and ovarian cancer initiation. These results explain why certain somatic driver mutations detected in normal tissues may not predict malignant progression, and that early detection strategies should instead prioritize testing deletions as potential biomarkers.

**Funding:**

10.13039/100000002NIH/10.13039/100000054NCI (P30CA016042; 1U01CA214194-01), NIH NIGMS (R35GM138113, 2R35GM138113), ACS (RSG-22-115-01-DMC), CIHR Vanier Fellowship, and the Francis Crick Institute with core funding from Cancer Research UK, UK 10.13039/501100000265Medical Research Council, and 10.13039/100010269Wellcome Trust.

**Research in context**

**Evidence before this study**

Classical multi-hit models and sequencing studies emphasize point mutations, but the role of copy number variations as tumour-initiating events was poorly defined.

**Added value of this study**

By integrating theoretical modeling, bulk sequencing, and single-cell analyses, we show that genomic deletions are the likely rate-limiting events in *BRCA1/2*-driven and sporadic breast and ovarian cancers.

**Implications of all the available evidence**

Deletions explains the heightened risks of *BRCA1/2* carriers and consistently arise as early, recurrent events across datasets and models, reshaping our understanding of tumour initiation. These findings highlight deletions as critical biomarkers for risk stratification and precision prevention, and challenge the sufficiency of point-mutation detection alone for driving tumourigenesis in breast and ovary tissues.

## Introduction

Cancer arises through the accumulation of somatic mutations,^1^^,^^2^ but the specific types of genomic variation and number of molecular alterations required for tumour initiation remain unclear. Several studies have identified accumulations of cancer-associated mutations in normal tissues,^3^^,^^4^ including in epithelial and stromal compartments of breast tissues in healthy adults.^5^ Further, large numbers of pre-malignant lesions are diagnosed each year and must be risk-stratified to identify their likelihood of progression to aggressive cancers. Thus, delineating the rate-limiting steps of tumourigenesis remains a critical unresolved problem in cancer biology.

Both theoretical and observational approaches have been taken to resolve this question.^6^, ^7^, ^8^, ^9^, ^10^, ^11^, ^12^, ^13^, ^14^ Theoretical models relating the number of mutations in driver genes to age-at-diagnosis distributions suggest 2–10 driver mutations are required, based on differing assumptions on drivers, pathways, and environmental influences.^6^, ^7^, ^8^, ^9^ Observational approaches have been developed to distinguish somatic driver mutations from background passenger alterations, including somatic recurrent point mutations,^10^ positively-selected point mutations,^11^ aberrant methylation,^12^ genomic rearrangements^13^ and somatic copy number aberrations (SCNAs).^14^

Seminal studies by Nordling^15^ and Armitage and Doll^16^ demonstrated that the number of aberrations required for tumourigenesis could be inferred from the relationship between age and cancer incidence. Knudson famously compared the incidence curves for familial and sporadic retinoblastoma, resulting in the “two-hit” hypothesis for *RB1*,^17^ and the more recent discovery of “one-hit” early-onset retinoblastoma.^18^ Tomasetti and colleagues extended this framework to estimate driver count from the differential mutation and incidence rates between two cancer sub-groups.^9^ They leveraged differing somatic single nucleotide variant (SNV) mutation rates estimated from DNA sequencing and epidemiological incidence rates to estimate the minimum number of sequential SNVs required to initiate a cancer. Comparing smokers *vs.* non-smokers in lung cancer and microsatellite instable (MSI) *vs*. microsatellite stable (MSS) colorectal cancer, they identified a minimum of three sequential SNVs were required for both diseases.

Carriers of pathogenic germline *BRCA1* and *BRCA2* variants are strongly predisposed to cancers in multiple organs, including prostate, pancreatic, breast and ovarian cancer.^19^, ^20^, ^21^ Similar to lung tumours in smokers and MSI colorectal tumours–tumours in *BRCA1* and *BRCA2* carriers show elevated mutation rates,^22^, ^23^, ^24^, ^25^, ^26^ at least in part a result of their homologous recombination deficiency (HRD).^27^ Women with pathogenic germline variants in these genes are often intensively screened, and pre-malignant lesions are common in both the breast and ovaries.^28^, ^29^, ^30^, ^31^ To quantify the rate-limiting mutational processes in the initiation of these cancers, we developed a quantitative driver-estimation framework that extended statistical formulations by Tomasetti et al.^9^ to estimate the minimum number of SNV, short insertions and deletion (INDEL) and SCNA drivers by comparing groups with differential mutation rates. We applied this framework to 3236 breast and ovarian sporadic *vs.* germline *BRCA1/2*-driven cancer cases, identifying deletions as the rate-limiting mutational process leading to breast and ovarian tumourigenesis—contrary to the emphasis placed on point mutations in these tumours. We confirm these observations using both driver prioritization^10^^,^^32^ and reconstruction of clonal evolution.^33^ Taken together, we provide a new framework for integrating theoretical and empirical approaches to driver mutation requirements across all types of driver mutation. We demonstrate its utility for HRD-cancers, informing early-detection and risk-stratification approaches in breast and ovarian cancer.

## Methods

### Datasets

#### TCGA germline *BRCA1* and *BRCA2* status in breast and ovarian cancer

Patients with pathogenic variants in *BRCA1* and *BRCA2* were identified by the TCGA PanCanAtlas Germline project.^34^ These patients did not have pathogenic germline variants in established HRD-related genes, including *ATM*, *CHEK2*, *PALB2*, *BARD1*, *RAD51C*, *RAD51D*, and *ATR*. To avoid confounding of germline and somatic differences by ancestry,^35^^,^^36^ we only considered individuals of European descent.

#### TCGA sample somatic mutation rate

We downloaded the public MAF somatic mutation call file from the TCGA PanCanAtlas MC3 project^37^ that conducted using a variety of variant calling tools. We then filtered the file to retain only non-synonymous mutations (in one of these types: “Missense_Mutation”, “Nonsense_Mutation”, “Splice_Site”, “Translation_Start_Site”). The mutation rate is calculated as the number of non-synonymous mutations divided by the age at the initial diagnosis for each sample. For somatic *TP53* mutation status, we only considered the nonsynonymous mutations, including missense, non-sense, frameshifting, in-frame shifting, or splice-site altering single-nucleotide changes or indels. As described previously,^38^^,^^39^ *TP53* truncations or missenses predicted as functional by at least one algorithm were considered.

#### TCGA sample copy-number variation rate

We downloaded the ABSOLUTE-annotated seg file from the TCGA PanCanAtlas Aneuploidy project^40^ (https://gdc.cancer.gov/about-data/publications/pancan-aneuploidy). SCNA burden was calculated as both the number of distinct segments and the number of base-pairs involved in a SCNA. Both measures were normalized by the age of initial diagnosis for each sample. Segments and altered bases were further categorized as being involved in a gain or a deletion event.

#### TCGA sample small INDEL mutation rate

We downloaded the public MAF somatic mutation call file from the TCGA PanCanAtlas MC3 project^37^ that leveraged a variety of variant calling tools, including Mutect, Somatic Sniper, Varscan and Pindel, as previously described. We then filtered the file to retain only small insertions and deletions. The INDEL burden is calculated as both the total number of distinct INDELs and the total number of base pairs in an INDEL divided by the age at the initial diagnosis for each sample.

#### METABRIC *BRCA1* and *BRCA2* status in breast cancer

Patients with pathogenic germline variants, defined as non-synonymous, frameshift, stop/gain or impacting splicing, in *BRCA1* and *BRCA2* were identified in 1980 breast tumours from METABRIC.^41^

#### METABRIC sample somatic mutation rate

Somatic single nucleotide variants were detected from targeted sequencing of 173 genes as described previously.^42^ The mutation rate is calculated as the number of non-synonymous mutations divided by the age at the initial diagnosis for each sample.

#### METABRIC sample copy-number variation rate

Copy number segments were identified from Affymetrix SNP 6.0 as described previously.^41^ SCNA burden was calculated two ways: the number of base-pairs or the number of genes involved in a SCNA. Both measures were normalized by the age of initial diagnosis for each sample. Altered bases and genes were further categorized as being involved in a gain or a deletion event.

#### Standardized incidence ratios for breast and ovarian cancer

We performed an extensive literature search for standardized incidence ratios (SIRs) for both breast and ovarian cancer amongst carriers of *BRCA1* and *BRCA2*. We utilized SIR estimates derived by Kuchenbaecker et al. for a variety of reasons.^43^ The multinational cohort used to calculate these SIRs was the largest amongst all studies reviewed, the range of ages represented was greater than most, and the study was one of the most recent [Supplementary Table S1]. The estimates listed below are total SIRs through age 80.

Breast Cancer:

Total SIR (95% CI) *BRCA1*: 16.6 (14.7–18.7)
Total SIR (95% CI) *BRCA2*: 12.9 (11.1–15.1)

Ovarian Cancer:

Total SIR (95% CI) *BRCA1*: 49.6 (40.0–61.5)
Total SIR (95% CI) *BRCA2*: 13.7 (9.1–20.7)

#### Incidence ratio estimation

We estimated the incidence ratio using a framework proposed by Tomasetti et al.^9^ Briefly, Tomasetti et al. showed there is a power relationship between cancer incidence and the number of rate-limiting mutational events for the initiation of a cancer subgroup and the average mutation rate of the cancer subgroup.^9^ For example, a cancer subgroup that has a mutation rate twice that of another subgroup will have an incidence rate 2^n^ that of the second subgroup, where n represents the number of rate-limiting mutational events required for tumourigenesis. Here we consider *BRCA1* or *BRCA2* carriers compared to sporadic breast and ovarian cancers.

Non-carrier breast and ovarian cancer samples were matched to *BRCA1* or *BRCA2* carrier samples separately, ensuring equal sample sizes within each cancer type and carrier status. We performed this matching using a weighted random sampling procedure, implemented via custom scripts in R. For breast cancer, non-carrier tumours were matched based on the distribution of PAM50 subtypes (Luminal A, Luminal B, *HER2*-enriched, Basal-like, Normal-like), pathologic stages (I-IV), and somatic *TP53* driver status, to match the sample size of *BRCA1* or *BRCA2* carriers. For ovarian cancer, matching was performed based on clinical stage distribution and somatic *TP53* driver status. To minimize potential confounding by other germline predispositions to HRD, we filtered out and validated that the WT/non-carrier individuals did not carry pathogenic or likely pathogenic germline variants in established HRD-related genes, including *ATM, CHEK2, PALB2, BARD1, RAD51C, RAD51D*, and *ATR*. Next, we calculated the ratio between the median non-carrier mutation rate and the median *BRCA1* or *BRCA2* carrier mutation rate. Finally, we estimated the incidence ratio considering the mutation rate ratio, u, and various number of driver events, k, using the following:

$$\text{Incidence}\;\text{ratio}=u^{k}$$

Because each additional driver event is likely to add a growth advantage and assuming this growth advantage is constant with each successive driver, as done in Tomasetti et al.^9^ based on the modeling of self-renewal phase of a tissue^44^ and tumour clonal expansion,^45^ the incidence ratio prediction becomes:

$$x\cdot x^{\left(\frac{\lambda_{1}}{\lambda_{1}}\right)}\cdot x^{\left(\frac{\lambda_{1}}{\lambda_{2}}\right)}...\cdot x^{\left(\frac{\lambda_{1}}{\lambda_{\left(n-1\right)}}\right)}$$

where: $\frac{\lambda_{1}}{\lambda_{2}}=\frac{1}{2},\frac{\lambda_{1}}{\lambda_{3}}=\frac{1}{3},etc$; x represents the relative mutation rate between two groups, such as *BRCA1* carriers and non-carriers. The λ terms describe the relative growth advantage conferred by each successive driver mutation during tumourigenesis. These growth advantages contribute to the overall increase in cancer incidence as mutations accumulate. We repeated these randomly sampling 10,000 times and compared the median across the 10,000 iterations to the observed SIRs. We considered the driver number that minimized the Kolmogorov–Smirnov metric between the estimated and observed SIR distribution to be the required number of drivers. We concluded a model failed to converge if the Kolmogorov–Smirnov metric never reached a minimum after testing 15 drivers. We repeated the same process with patients of ovarian cancer. We considered mutation rates based on SNVs, SCNAs, SCNA deletions, SCNA gains and INDEL deletions. For SCNAs and INDELs, we calculate the total number of bps involved in these aberrations.

A linear model for somatic SNV burden *vs.* age is justified by multiple independent datasets showing approximately constant, clock-like accrual per cell across tissues and species. Whole-genome sequencing of human adult stem-cell–derived organoids (colon, small intestine, liver) demonstrated linear increases up to thousands of mutations by late life with stable spectra, consistent with steady processes (notably SBS1/SBS5).^46^ In hematopoiesis, single-cell and clonal analyses found point mutations accumulate linearly through the lifespan at ∼16–17 substitutions per HSC/MPP per year, reinforcing a constant per-cell rate.^47^ Post-mitotic neurons also show an approximately linear rise in sSNVs with age, supporting a time-proportional model even in non-dividing cells.^48^

#### Mutational timing analysis

We leveraged mutational timing estimates from Gerstung et al.^33^ Briefly, for 28 and 26 recurrent somatic events (defined as occurring in >5% of samples) in breast and ovarian cancer, respectively, Gerstung et al. generated a discrete multinomial distribution based on pairwise probabilities one mutation occurs before another mutation, (x_i_, x_j_) ∼ p1, p2, p3.

$$p1=P\left(X_{i}\;\text{before}\;X_{j}\right)$$

$$p2=P\left(X_{j}\;\text{before}\;X_{i}\right)$$

$$p3=P\left(\text{order}\;\text{of}\;\left(X_{i},X_{j}\right)\text{unknown}\right)$$

Using league modeling, they stimulated games by drawing from this multinomial distribution and assigning points based on “wins”, *i.e.,* mutation A comes before mutation B, or “ties”, *i.e.,* the order of mutations unknown. The final scores were ranked to generate relative timing estimates, and this process was repeated at least 1000 times to obtain distributions of the rankings. Here, these relative timing estimates were compared to the recurrence of the mutation across samples of the same cancer type within the PCAWG cohort.

#### SCNA frequency maps

To generate the SCNA frequency maps of *BRCA1*, *BRCA2*, and non-carrier tumours, TCGA SNP array data was downloaded from the GDC repository (https://dcc.icgc.org/releases) genome version GRCh38. Data category ‘Masked Copy Number Segment’ derived from SNP6.0 genotyping arrays. SCNA frequency maps are generated using the R package svpluscnv (https://github.com/ccbiolab/svpluscnv) with the following options SCNA.freq (x, fc.pct = 0.2), where ‘x’ is the object containing segmentation data and ‘fc.pct’ is the percentage of SCNA log-R ratio change threshold defining gain and loss of copy number. i. e SCNA gain (red) threshold is logR > log2 (1+ fc.pct) and loss (blue) is logR < log2 (1–0.2). The same measures were used to determine chromosome arm SCNA frequencies reported samples with at least 30% of the chromosome arm length gained or lost.

#### Single-cell whole genome sequencing (scWGS) datasets and analyses

We analyzed three distinct datasets from Funnell, T. et al.^49^ that conducted single-cell whole genome sequencing (scWGS) on genetically engineered Mammary Epithelial Cells, triple-negative breast cancer (TNBC), and High-Grade Serous Ovarian Cancer (HGSC) datasets. The 184-hTERT Mammary Epithelial Cells datasets included clonal populations with genetically engineered *BRCA1*, *BRCA2*, and *TP53* deletions produced via CRISPR–Cas9 editing of 184-hTERT L9 cell lines: SA1054 (*BRCA1*^*−/−*^) with 382 cells, SA1055 (*BRCA2*^*−/−b*^) with 391 cells, SA1056 (*BRCA2*^−/−a^) with 496 cells, SA1292 (*BRCA1*^*+/−*^) with 377 cells, SA1188 (*BRCA2*^*+/−*^) with 472 cells, SA906a (*TP53*^*−/−a*^) with 650 cells, SA906b (*TP53*^*−/−b*^) with 984 cells, and SA039 (WT) with 878 cells. The TNBC dataset includes 4 FBI-dup samples, 2 HRD samples, and 1 TD sample. The HGSC dataset contained 8 FBI-dup samples, 6 HRD samples and 2 TD samples. In the CRISPR-edited cell lines, the experimental design of Funnell et al. mitigates the impact of potential CRISPR off-target effects that could affect our analyses. (1) All cell lines in this study were derived from single-cell clones, so that any CRISPR-induced genomic alterations would be present in all cells within each clone, making such alterations irrelevant to our analysis of cell-to-cell variation in chromosomal instability. (2) Following transient CRISPR editing, cells were extensively passaged before single-cell whole-genome sequencing was performed. This extended culturing period ensures that the analysis captures ongoing chromosomal instability rather than immediate artifacts from the CRISPR editing process. The lineage history, when mutations were introduced and when cells were sequenced are shown in Fig. 1a from Funnell et al. (3) Third, the initial study team validated all targeted mutations through both Sanger sequencing confirming that the CRISPR guides targeted the correct genomic regions. Supporting evidence, including Sanger traces are provided in Extended Data Fig. 2a from Funnell et al.

**Fig. 1:**
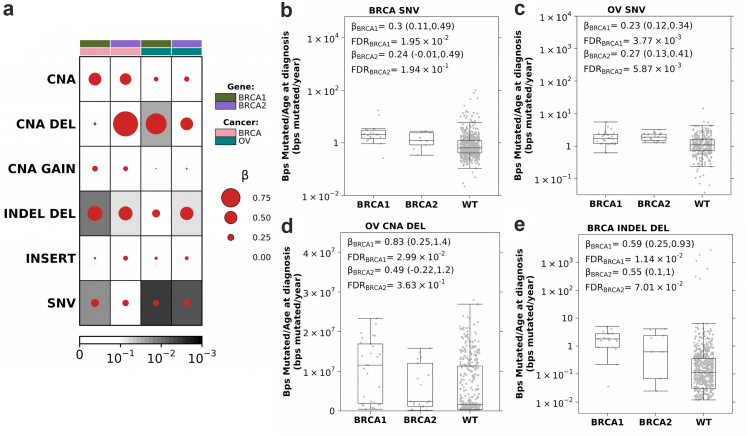
**Increased mutation burden in germline *BRCA1* and *BRCA2* carriers**. **(a)** Breast and ovarian tumours in *BRCA1* and *BRCA2* carriers show an increased mutation rate (bp/year) compared to non-carrier (WT) individuals. Dot size and colour reflect β magnitude and direction from linear regression correcting for stage, grade and subtype. Background shading indicates FDR. Covariate along the top indicates gene and cancer type. **(b and c)** Boxplots show increased coding SNV mutation rate (bp/year) in breast **(b)** and ovarian **(c)** tumours in *BRCA1* and *BRCA2* carriers compared to WT. Boxplots represent median, 0.25 and 0.75 quantiles with whiskers are 1.5× interquartile range. β and FDR from linear regression. **(d)***BRCA1* and *BRCA2* carriers show an increased SCNA deletion rate compared to WT in ovarian tumours. **(e)***BRCA1* and *BRCA2* carriers show an increased small deletion rate compared to WT in breast tumours. NV: single nucleotide variants; SCNA: copy number aberrations; DEL: deletions; INDEL: small insertion and deletions.

**Fig. 2:**
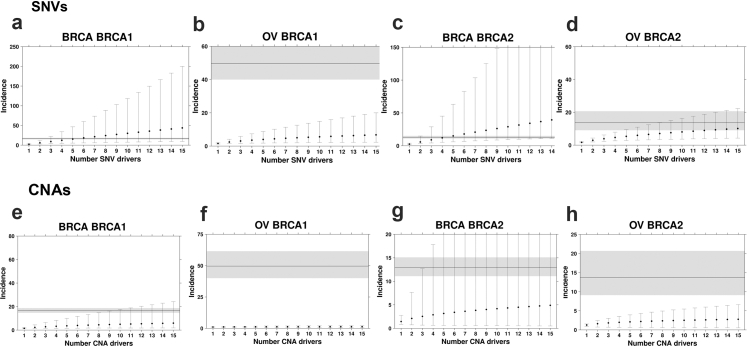
**SNV and SCNA mutation burden alone cannot explain breast and ovarian cancer incidence in germline *BRCA1* and *BRCA2* carriers**. Estimated incidence rates based on SNV **(a–d)** and SCNA **(e–h)** mutational burden with the increasing number of drivers cannot explain the observed incidence ratio of breast and ovarian cancer in *BRCA1* and *BRCA2* carriers. Points represent estimated incidence ratios based on the number of driver events along the x-axis for breast cancer in *BRCA1***(a and e)** and *BRCA2***(b and f)** carriers and ovarian cancer in *BRCA1***(c and g)** and *BRCA2***(d and h)** carriers. Error bars indicate 95% confidence intervals. The horizontal line indicates observed incidence rate, and grey shading indicates 95% confidence intervals. Incidence ratios for carriers were benchmarked against non-carrier controls matched for tumour subtype, stage, and somatic *TP53* status.

Based on the scWGS data, SCNA segment sizes were calculated in base pairs and converted to megabases (MB) affected by copy number deletions and SCNA gains, respectively, and subsequently the median fold changes for these SCNAs and SNVs across different groups were calculated using R. The Wilcoxon Rank Sum test was employed to statistically discern differences in SCNA and SNV distributions across groups or phenotypes. P values were adjusted for multiple testing using the BH procedure for FDR.

Additionally, a total of 15 luminal breast epithelial samples from Williams et al.^50^ were included for analysis as these samples had allele-specific copy number estimates. These comprised benign tissues from germline *BRCA1* carriers (B1-43, B1-49, B1-6410, B1-6537, B1-6550), *BRCA2* carriers (B2-18, B2-25, B2-6532), and wild-type individuals (WT-6, WT-62), along with precancerous lesions including one ductal carcinoma in situ (B2-18), one lobular carcinoma in situ (B2-23), and two atypical lobular hyperplasias (B2-21, WT-6752). One benign sample (B1-6548) carried both germline *BRCA1* and *BRCA2* alterations. SCNA segment sizes were calculated in base pairs, converted to megabases, and compared across groups using the same pipeline as for prior scWGS datasets. Analyses to identify cells with *BRCA1/BRCA2* somatic deletions used the R packages BioMart and GenomicRanges.

#### Single-cell RNA-seq (scRNA-Seq) dataset and analysis

Seurat objects of single-cell RNA-seq data were obtained from human breast tissue from 21 patients with no history of cancer treatment as previously described.^49^^,^^51^ This scRNA-Seq dataset was generated with the 10× Genomics Chromium platform and an Illumina NextSeq sequencer. The samples were taken from patients at different stages of cancer progression, including normal healthy cell samples as well as various tumour samples.

The paper provided data on triple-negative breast cancer (TNBC) tissues of germline *BRCA1* carriers *vs.* non-carriers, as well as preneoplastic and normal breast tissue. TNBC taken from four *BRCA1* carriers were labeled “B1”. The other TNBCs were labeled “TN”. The seurat object containing both samples, *SeuratObject_TNBC.rds*, was further divided into four *BRCA1* variant samples and four non-*BRCA1* variant samples. The 1 normal premenopausal epithelial breast tissue sample (N_1105_epi) used as a reference sample was from the *SeuratObject_NormEpi.rds* object. Data from precancerous and pathologically normal breast tissue was sourced from the seurat object *SeuratObject_NormB1Total.rds*. Lastly, the four samples of preneoplastic normal breast tissue that was taken from four *BRCA1* variant carriers were labeled “PN” and the non-carrier premenopausal normal breast tissue samples were labeled “TP”.

Using the R package, inferCNV,^52^ we inferred SCNAs of *BRCA1* TNBCs, non-carrier TNBCs, and their respective normal counterparts. The inferCNV package analyzes the intensity of gene expression across the genome of a tumour sample and compares these values to a designated normal somatic cell sample as a reference. The i6 Hidden Markov Model (HMM) assigned six states of copy number variation to each tumour cell. The states 2 and 1 were used in the creation of violin plots for deletion as they correspond to a loss of one and two copies, respectively. The same was done with SCNA gain as states 4 and 5 represent the gain of one and two copies. SCNA segment sizes were calculated in base pairs and converted to megabases (MB) affected by copy number deletions and SCNA gains, respectively, and subsequently the median fold changes for these SCNAs across different groups were calculated. The Wilcoxon Rank Sum test was employed to statistically discern differences between groups, and P values were adjusted for multiple testing using the BH procedure for FDR.

#### Quantification of uncertainty and operating characteristics

We extended the incidence–mutation framework used in the main analysis to quantify uncertainty around the required number of driver events (k) and to evaluate operating characteristics (“power”) under current sample sizes. For each scenario (*e.g.*, SNVs, SCNAs, deletions) and stratum (BRCA1/2 in breast/ovary), we performed a double bootstrap: (i) resampling carrier/non-carrier tumors with replacement within the matched design to recompute the mutation-rate ratio (u), and (ii) sampling the standardized incidence ratio (SIR) on the log scale from a distribution parameterized to reproduce the published 95% CI. For each bootstrap replicate, we predicted incidence ratios across k = 1, …,K as uˆk (optionally applying a growth-advantage adjustment that divides by (k−1)!). We selected $\hat{k}$ by minimizing the absolute deviation between the predicted and sampled SIR. The median and 95% quantile interval of $\hat{k}$ across B replicates were reported as the point estimate and confidence interval. “Power” was quantified as the probability that the bootstrap procedure meets the pre-specified decision (*e.g.*, Pr [$\hat{k}$ ≤3]). Across B = 10,000 bootstrap replicates per scenario (K = 15), we obtained median k and 95% CIs and visualized the distributions (Supplementary Fig. S1a). With current cohort sizes (2,1 carriers per scenario), deletion-based models typically yield narrow intervals around low k and high probability that k ≤ 3, whereas SNV-only models are less precise—consistent with our primary conclusions.

#### Data visualization

Visualizations were generated in the R statistical environment (v3.3.1) with the lattice (v0.24–30), latticeExtra (v0.6–28) and BPG (v5.6.23) packages.^53^

#### Ethics

All data analyzed in this study were obtained from publicly available resources. Germline *BRCA1/2* variant calls and somatic mutation data for The Cancer Genome Atlas (TCGA) Pan-Cancer Atlas and METABRIC were accessed through the Genomic Data Commons and the PanCanAtlas Germline Project, with germline data obtained under approved dbGaP application. Somatic evolutionary timing data were accessed from the ICGC/TCGA Pan-Cancer Analysis of Whole Genomes (PCAWG) project. All original studies obtained informed consent from participants and were approved by their respective institutional review boards and ethics committees, as described in their primary publications. No new patient recruitment or experimental procedures involving human subjects were conducted in this study.

#### Statistics

All statistical analyses were performed in the R statistical environment using base functions and relevant packages for genomic data visualization and modeling. For comparisons of somatic alteration rates (SNVs, INDELs, SCNAs), we used linear regression models adjusted for potential confounders (tumour subtype, stage, and grade), reporting effect sizes (β) with 95% confidence intervals. To estimate the number of rate-limiting mutational events, we extended the incidence–mutation rate framework of Tomasetti et al., fitting observed incidence ratios against mutation burden ratios using bootstrapping (10,000 iterations) and minimizing the Kolmogorov–Smirnov (KS) statistic to identify the most parsimonious number of required drivers. For single-cell analyses, group differences in mutation burdens (SNVs, SCNAs) were assessed using the Wilcoxon Rank Sum test, with multiple testing correction via the Benjamini–Hochberg procedure.

##### Sample size determination

No formal sample size calculation was performed, as the study was based on available cohorts (TCGA, METABRIC, PCAWG, scRNA-seq, and scWGS datasets). The large size of these cohorts provided sufficient power for statistical inference.

##### Randomization

To avoid confounding, non-carrier control samples were matched to BRCA1/2 carriers using weighted random sampling stratified by clinical variables (tumour subtype, stage, TP53 mutation status).

##### Blinding and inclusion/exclusion criteria

Investigators were not blinded to carrier status during analysis, and no additional inclusion/exclusion criteria were applied, as all data were computationally derived from existing repositories.

#### Role of funders

The funding sources provided financial support for the researchers; the funders have no role in the study design, data analyses, or manuscript writing/editing.

## Results

### *BRCA1* and *BRCA2* carriers showed varied rates of somatic alterations

While the role of *BRCA1* and *BRCA2* in homologous recombination has been well characterized, the consequence of variants in these two genes is lineage dependent.^21^ We focus here on two canonical *BRCA1/2*-associated cancers—breast (BRCA) and ovarian (OV) cancers. Leveraging data from TCGA Pan-Cancer Atlas,^34^^,^^37^ we interrogated the somatic mutation landscape of 758 breast and 498 ovarian tumours, a subset of which harboured pathogenic germline variants in *BRCA1* (n_breast_ = 20; n_ovarian_ = 31) or *BRCA2* (n_breast_ = 12; n_ovarian_ = 22) as evaluated by CharGer and the PanCanAtlas germline study.^34^ Due to the limited sample sizes in non-Europeans, we only consider individuals of European descent to avoid confounding by ancestry-biased somatic mutations.^54^ There were no significant differences in tumour purity or ploidy between carriers and non-carriers (Supplementary Fig. S1b and c), aside from *BRCA2* loss-of-function ovarian samples, which had significantly lower tumour purity than non-carrier samples. The lower purity suggests our mutation estimates for this subgroup are conservative (because of reduced effective coverage) and thus serve as a lower bound.

We first compared the burden of diverse genomic alterations, including SNVs, SCNAs, and INDELs, in g*BRCA1* and g*BRCA2* carriers *vs.* non-carriers. SNVs and INDELs (<1kbp) were obtained from TCGA PanCanAtlas MC3 consensus mutation call sets based on DNA-Seq data, whereas SCNAs were obtained by TCGA PanCanAtlas ABSOLUTE-annotated SCNAs based on SNP-array data. As expected,^22^, ^23^, ^24^, ^25^ somatic SNV mutation counts, normalized by age at diagnosis and adjusted for stage, grade and subtype, were elevated in breast and ovarian tumours arising in individuals carrying pathogenic germline variants in *gBRCA1* (β_breast_ = 0.30 (95% Confidence Intervals (CI): 0.11–0.49); FDR_breast_ = 1.9 × 10^−2^; β_ovarian_ = 0.23 (95% CI: 0.12–0.34); FDR_ovarian_ = 3.8 × 10^−3^; linear regression) or *gBRCA2* (β_breast_ = 0.24 (95% CI: −0.01 to 0.50); FDR_breast_ = 0.19; β_ovarian_ = 0.27 (95% CI: 0.13–0.41); FDR_ovarian_ = 5.7 × 10^−3^; Fig. 1a–c).

Because SCNAs also accumulate with age,^55^ we considered the total number of base pairs involved in SCNAs, normalized by age at diagnosis. After controlling for stage, grade and subtype, there was no significant difference in SCNA burden between carriers and non-carriers (FDR >0.1; Fig. 1a). Considering gains (GAIN) and deletions (DEL) separately, ovarian tumours in *BRCA1* carriers had a significantly higher SCNA deletion burden than non-carriers (β = 0.83 (95% CI: 0.25–1.4); FDR = 3.0 × 10^−2^; Fig. 1a and d) while no significant difference in SCNA gains was observed between carriers and non-carriers (FDR ≥0.64; Supplementary Fig. S2).

Next, we considered INDELs (<1kbp) based on consensus calling by the TCGA PanCanAtlas MC3 project.^34^ We calculated INDEL burden as the number of base pairs involved in an INDEL normalized by age at diagnosis and similarly quantified a significant increase in INDEL deletions in *BRCA1* (β_breast_ = 0.59 (95% CI: 0.25–0.93); FDR_breast_ = 1.14 × 10^−2^; β_ovarian_ = 0.30 (95% CI: −0.02 to 0.62); FDR_ovarian_ = 0.19) and *BRCA2* (β_breast_ = 0.55 (95% CI: 0.10–1.0); FDR_breast_ = 6.5 × 10^−2^; β_ovarian_ = 0.52 (95% CI: 0.11–0.92); FDR_ovarian_ = 6.4 × 10^−2^) carriers (Fig. 1a and e). No significant increased burden of INDEL insertions was observed (FC ≤ 0.18; FDR ≥0.31; Supplementary Fig. S2). Thus, breast and ovarian tumours in *BRCA1* and *BRCA2* carriers recapitulate the elevated burden of diverse genomic alterations expected of this high-risk group.

### Accounting for increased incidence ratio of *BRCA* carriers

Expanding upon the insights of Nordling, Armitage and Doll,^15^^,^^16^ Tomasetti et al. showed cancer incidence is a function of the number of rate-limiting mutational events required for initiation of a cancer subgroup and the average mutation rate of that mutation type in that cancer subgroup.^9^ This framework assumes a constant mutation rate over evolutionary time. For example, a cancer subgroup that has a mutation burden twice that of another subgroup will have an incidence rate 2nd that of the second subgroup, where n represents the number of rate-limiting mutational events required for tumourigenesis (Methods). *BRCA1* and *BRCA2* are key components of the DNA damage repair pathway; their pathogenic germline variants lead to deficient homologous-directed repair. We hypothesize that the elevated incidence rates in *BRCA1* and *BRCA2* carriers can, in part, be explained by their increased mutational burden. We therefore estimated the number of driver events required to initiate breast and ovarian cancer by comparing *BRCA1* and *BRCA2* germline variant carriers to non-carriers (Methods). By expanding the framework to consider multiple somatic alteration types, we can quantify the number of each alteration type required to converge on the observed incidence rates and identify the rate-limiting mutational processes. Identifying the rate-limiting mutational processes shapes how we understand tumour evolution.

We first reviewed the epidemiology literature to obtain standardized incidence ratios (SIRs) of breast and ovarian cancer in *BRCA1* and *BRCA2* carriers compared to matched control populations (Supplementary Table S1). The best powered study, including 6036 *BRCA1* and *BRCA2* female carriers, revealed SIRs of: SIR_breast|*BRCA1*_ (95% CI): 16.6 (14.7–18.7), SIR_breast*BRCA2*_: 12.9 (11.1–15.1), SIR_ovarian|*BRCA1*_: 49.6 (40.0–61.5), SIR_ovarian|*BRCA2*_: 13.7 (9.1–20.7).^43^

**Fig. 3:**
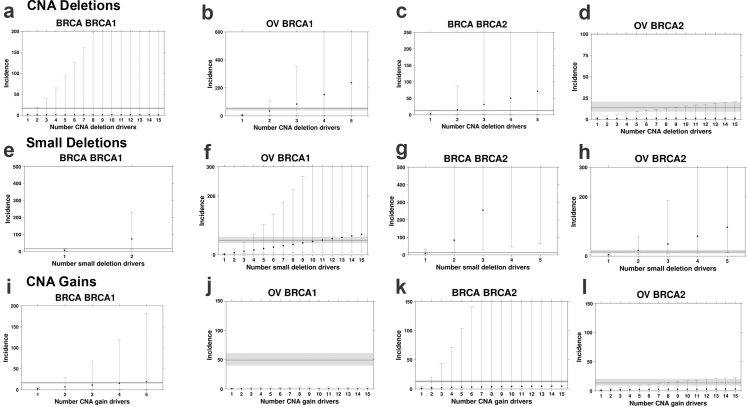
**Deletions are the likely rate-limiting mutational process in the breast and ovarian tumours of germline *BRCA1* and *BRCA2* carriers**. **(a–d)** Incidence estimates based on deletion rates converge on the observed incidence rate. Deletion burden calculated as the number of base pairs altered normalized by age at diagnosis. **(e–h)** Incidence estimates based on small deletions. **(i–l)** Incidence estimates based on gain rates. Segplots compare estimated incidence rate to observed incidence rate as described in Fig. 2.

Next, we conducted a bootstrapped estimation (n = 10,000) of the average increase in SNV mutation burden (*i.e.,* protein-altering SNV counts normalized by age at diagnosis) for *BRCA1* and *BRCA2* carriers compared to clinically matched non-carrier individuals with breast and ovarian cancer. The non-carrier individuals were matched to carriers based on the distribution of relevant factors:, including pathological stage, somatic *TP53* mutation status, and PAM50 subtype (for breast cancer only) (Methods, Supplementary Table S2). We identified the expected increase in normalized SNV counts in carriers (median FC_breast|*BRCA1 vs.* WT_ (95% CI) = 3.57 (2.17–5.19); median FC_breast|*BRCA2 vs.* WT_ = 2.21 (1.71–4.06); median FC_ovarian|*BRCA1 vs.* WT_ = 1.60 (1.13–2.16); median FC_ovarian|*BRCA2 vs.* WT_ = 1.82 (1.31–2.61); Supplementary Fig. S3a). Finally, we compared the estimated mutation burden ratios to observed SIRs for each pair of germline risk-gene and cancer type (Figs. 2 and 3). For breast cancer, *BRCA1* carriers had 2.84-fold more protein-altering SNVs than non-carrier patients. Thus, if breast cancer initiation in these individuals required two driver SNVs, the estimated incidence ratio would be 2.84^2^ = 8.06. If three coding SNV drivers are required, the estimated incidence ratio would be 3.57 × 3.57 × 3.57^1/2^ = 13.6 (see Methods). The observed SIR for breast cancer *BRCA1* carriers is 16.6; therefore, the framework estimated a minimum of five SNVs are required to initiate breast cancer in *BRCA1* carriers (D = 0.43 (0.424–0.445; p < 0.001; Supplementary Table S3); Kolmogorov–Smirnov test; Fig. 2a). The framework estimates four SNV drivers are required to initiate breast in *BRCA2* carriers (D_breast_ (4 drivers) = 0.45 (0.445–0.465; Supplementary Table S3); Kolmogorov–Smirnov test; Fig. 2c), however, it fails to converge on the ovarian SIR in *BRCA1* and BRCA2 carriers (Kolmogorov–Smirnov D did not reach minimum; Fig. 2b and d). These data suggest the SNV mutation burden does not fully capture the mechanisms of germline *BRCA1* and *BRCA2* deficiency, and SNVs may not be the sole rate-limiting mutational process leading to tumourigenesis in *BRCA1* and *BRCA2* carriers.

Next, we explored other mutational processes that may explain the increase in cancer incidence in *BRCA1* and *BRCA2* carriers. Given that ovarian tumours are C-class tumours characterized by high SCNA rates,^14^ we evaluated whether elevated SCNA burden, *i.e., SCNA* bp/year, could explain the incidence of breast and ovarian cancer in *BRCA1* and *BRCA2* carriers. Compared to WT, *BRCA1* and *BRCA2* carriers had modest increases in SCNA burden (median FC_breast|*BRCA1*_ _*vs.*_ _WT_ (95% CI) = 1.26 (1.00–1.54); median FC_breast|*BRCA2 vs.* WT_ = 1.06 (0.83–1.76); median FC_ovarian|*BRCA1 vs.* WT_ = 1.19 (1.06–1.37); median FC_ovarian|*BRCA2 vs.* WT_ = 1.15 (1.03–1.34); Supplementary Fig. S3b). Our framework failed to reach the observed SIR using either g*BRCA1* or g*BRCA2* in breast or ovarian cancers (Fig. 2e–h). Driver estimates were similar when calculating SCNA burden as the number of SCNA segments normalized by age at diagnosis, the framework converges only for breast cancer in *BRCA2* carriers and estimates that twelve SCNA drivers are required to initiate breast cancer in *BRCA2* (Supplementary Fig. S4d). An SCNA driver could be a single event that deletes or amplifies multiple genes in proximity, *e.g.*, *RB1* and *BRCA2* on chromosome 13, or a single event capturing a single driver gene and many passenger genes.

Pathogenic *BRCA1* and *BRCA2* variants induce homologous recombination deficiency (HRD), which has been associated with increased rates of genomic deletions.^13^^,^^23^^,^^56^ Thus, we next considered deletions and gains separately. Considering only deletions and calculating mutation burden based on the number of deletions or base pairs altered, the ratio of SCNA mutation rates converged on the observed SIR in both ovarian and breast cancer (Fig. 3b and c; Supplementary Fig. S4g) (Supplementary Table S3). *BRCA1* and *BRCA2* carriers had increased deletion mutation rates (bp/year) compared to non-carrier individuals (median FC_breast|*BRCA1 vs.* WT_ (95% CI) = 1.18 (0.28–6.46); median FC_breast|*BRCA2 vs.* WT_ = 4.48 (0.83–37.6); median FC_ovarian|*BRCA1 vs.* WT_ = 6.97 (1.26–17.6); median FC_ovarian|*BRCA2 vs.* WT_ = 1.80 (0.32–10.6); Supplementary Fig. S3c). Modeling based on SCNA deletions estimated two to four deletions might be needed to initiate ovarian or breast cancer in *BRCA1* and *BRCA2* carriers (D_ovarian|*BRCA1*_ (2) = 0.45 (0.446–0.465); D_breast|*BRCA2*_ (2) = 0.43 (0.429–0.449)) (Fig. 3b and c). Modeling based on deletion segments rather than base pairs showed convergence for *BRCA2* in breast cancer (D_breast|*BRCA2*_ (4) = 0.44 (0.431–0.450)) but not others (Supplementary Fig. S4; Supplementary Table S3).

Next, we considered small deletions (<1kbp) and observed an increased rate of small deletions (bp/year) in the *BRCA1* and *BRCA2* carriers compared to non-carrier individuals (median FC_breast|*BRCA1 vs.* WT_ (95% CI) = 17.4 (6.60–41.5); median FC_breast|*BRCA2 vs.* WT_ = 6.98 (0.42–33.7); median FC_ovarian|*BRCA1 vs.* WT_ = 3.00 (1.16–7.08); median FC_ovarian|*BRCA2 vs.* WT_ = 4.62 (1.41–14.8); Supplementary Fig. S3d). Based on small deletions, considering both the number of deletions and base pairs altered, we estimate that less than ten small deletions are required for breast or ovarian tumourigenesis in *BRCA1* and *BRCA2* carriers (D_breast|*BRCA1*_ (1) = 0.48 (0.472–0.492); D_ovarian|*BRCA1*_ (10) = 0.45 (0.447–0.467); D_breast|*BRCA2*_ (1) = 0.44 (0.436–0.456); D_ovarian|*BRCA2*_ (2) = 0.48 (0.473–0.493); Fig. 3e–h) (Supplementary Table S3). In contrast, *BRCA1* and *BRCA2* carriers did not show a consistent increase in gain rates (median FC_breast|*BRCA1 vs.* WT_ (95% CI) = 4.19 (0.90–10.4); median FC_breast|*BRCA2 vs.* WT_ = 1.46 (0.26–4.71); median FC_ovarian|*BRCA1 vs.* WT_ = 0.37 (0.25–1.38); median FC_ovarian|*BRCA2 vs.* WT_ = 1.14 (0.30–3.52); Supplementary Fig. S3e). Modeling based on SCNA gains estimated that four drivers are required in *BRCA1*-driven breast cancer (D (4) = 0.42; 0.4110.431, Supplementary Table S3; Fig. 3i), however, the framework failed to reach the observed SIR for BRCA2 in breast cancer and both genes in ovarian cancer (Kolmogorov–Smirnov D did reach a minimum; Fig. 3j–l). Driver estimates were similar when calculating SCNA gain burden as the number of SCNA gain segments normalized by age at diagnosis (Supplementary Fig. S4m–p). Due to the large number of samples with no small insertions, the framework could not be applied to small insertions with the current sample sizes.

It is possible that loss of heterozygosity (LOH), accompanied by pathogenic germline *BRCA1/2* variants, is required to trigger loss of function in HRD and trigger tumourigenesis. Thus, we conducted another round of analyses using only *BRCA1* or *BRCA2* carriers showing LOH compared to non-carriers. For both breast and ovarian cancer, the differences in small deletion rates almost always amount to the least number of drivers for increased cancer incidence in *BRCA1* and *BRCA2* carriers—often less than two to three. This is except for modelling carriers in OV, where SCNA deletions require less than 3 drivers and small deletions require ∼15 drivers. Regardless, the framework converged less effectively for SNVs or amplifications as potential drivers, confirming our prior findings (Supplementary Figs. S5 and S6).

To verify our results, we replicated this statistical analysis in the METABRIC cohort of 1980 breast tumours,^41^ which included 26 *BRCA1* and 31 *BRCA2* carriers (Methods). The framework failed to reach the observed SIR when considering total SNV and SCNA burden (Supplementary Fig. S7). Only when considering the number of genes lost via SCNA deletion did the model converge for both *BRCA1*-and *BRCA2*-driven breast cancer, aligned with the observation in TCGA. Our model predicted 4 and 9 gene drivers were required in *BRCA1* (D (4) = 0.42 (0.411–0.431) and *BRCA2* (D (9) = 0.44 (0.430–0.450; Supplementary Table S3) driven tumors, respectively.

**Fig. 4:**
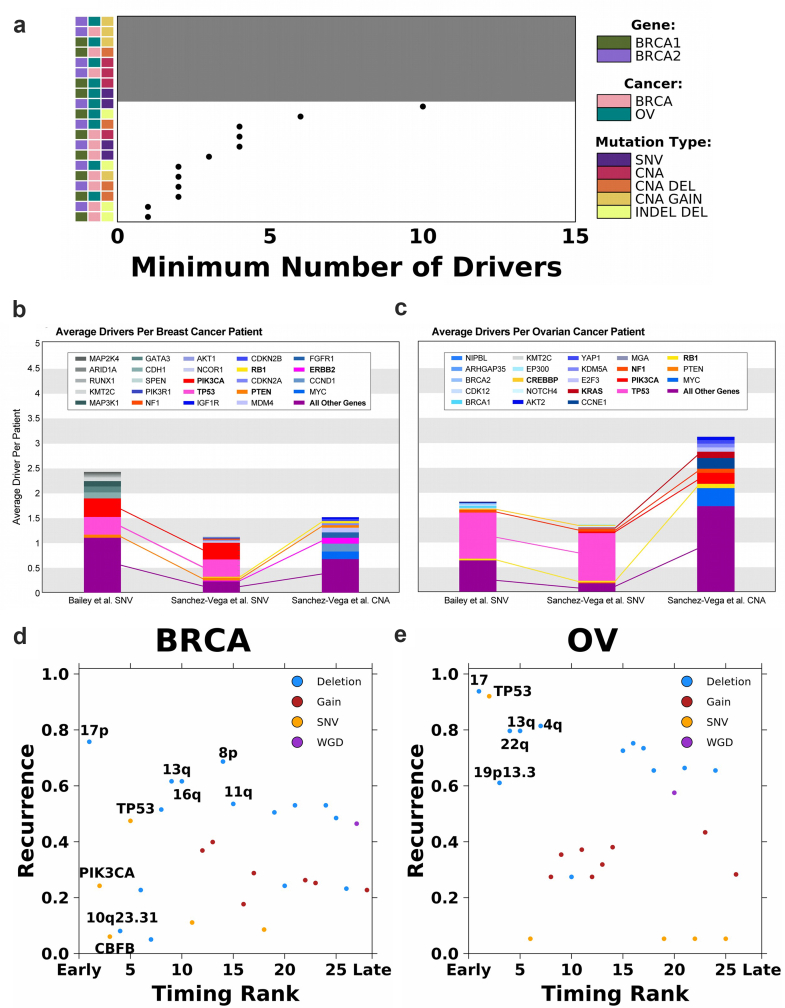
**Empirical driver and timing prediction from sequencing studies**. **(a)** Summary of the minimum number of drivers estimated for each cancer type, carrier and mutation type modeled. Grey background indicates the model did not converge and covariate along the left indicates cancer type, carrier and mutation type modeled. **(b and c)** The average number of drivers per patient observed for each gene from sequencing data in TCGA breast and ovarian cancers. The top 10 genes with the highest average driver count per patient are colored distinctively, and all other driver genes are grouped into a single category. Connections between stacked bars indicate the same genes in the top 10 across the driver prioritization method and variant type. Each stacked bar signifies the average total driver SNV or SCNA found amongst patients with breast **(b)** and ovarian **(c)** cancers. **(d and e)** Recurrence (y-axis) and relative timing (early *vs.* late; x-axis) for 28 and 26 drivers in breast **(d)** and ovarian **(e)** cancer, respectively. Drivers are colored based on the type of mutational event. The union of the top five most recurrent and earliest occurring drivers are labeled.

These data suggest deletions are likely the predominant rate-limiting mutational process leading to breast and ovarian tumourigenesis if tumours are initiated based on the least numbers of required drivers (Fig. 4a). However, an assumption of our framework is that a mechanism by which *BRCA1* and *BRCA2* germline variants increase the risk of tumourigenesis is through suppression of DNA damage repair leading to increased mutational burden.^22^, ^23^, ^24^, ^25^, ^26^ If *BRCA1* and *BRCA2* tumours have different driver type requirements than non-carrier tumours, comparison of alteration rates may be invalid. To evaluate the possibility, we compared SCNA patterns of *BRCA1*, *BRCA2* and non-carrier tumours for both cancer types (Methods). Breast and ovarian *BRCA1* and *BRCA2* tumours have higher frequencies of SCNAs but carriers had the same SCNA patterns as non-carrier tumours (Supplementary Fig. S8). For example, the most recurrent SCNAs in breast cancer include chromosome 17p deletion (frequencies in *BRCA1*: 30.0%; *BRCA2*: 63.6%; WT: 37.7%) and chromosome 8q gain (frequencies in *BRCA1*: 70.0%; *BRCA2*: 72.7%; WT: 45.8%). The most recurrent SCNAs in ovarian cancer include 8q gain (frequencies in *BRCA1*: 79.3%; *BRCA2*: 75.4%; WT: 62.7%). The similar SCNA landscapes suggest *BRCA1*, *BRCA2* and non-carrier tumours require similar deletions and gains while acquiring these alterations at different rates. Both BRCA-driven hereditary and sporadic tumours evolve to converge on a similar tumourigenic state. Based on these results, we concluded the key assumption of our framework is supported.

### Comparison to genomic drivers prioritized using other methods

Existing methods mostly estimate drivers from either theoretical calculations or observed sequencing data, and few studies directly compared estimates of total driver counts derived from these methods.^57^ To offer an observational comparison, we derived the average driver gene SNV and SCNA profile for 1070 breast and 566 patients of ovarian cancer in TCGA using two driver prioritization methods described in Bailey et al.^10^ and Sanchez-Vega et al.^32^ Briefly, both methods focused on focal changes in known cancer driver genes. The prioritized SCNAs considered only recurrent SCNA gains or deep (possibility homozygous) deletions and were further required to correlate with mRNA abundance changes.

Fig. 4b and c shows the genes with the highest average SNV and SCNA driver count per patient. The average total SNV driver count was higher for both breast cancer (Fig. 4b) and ovarian cancer (Fig. 4c) using Bailey et al.‘s prioritization method^10^ compared to Sanchez-Vega et al.‘s prioritization method^32^ (n_breast_: 2.43 *vs.* 1.13; n_ovarian_: 1.80 *vs.* 1.35; Fig. 4b and c). As expected, both driver prioritization methods identify, on average, a minimum of one to three SNV drivers per patient. Both gene prioritization methods revealed *TP53* (Freq_Bailey *et al.*_: 0.36; Freq_Sanchez-Vega *et al.*_: 0.35; Fig. 4b) and *PIK3CA* (Freq_Bailey *et al.*_: 0.37; Freq_Sanchez-Vega *et al.*_: 0.34; Fig. 4b) to be the two most commonly mutated driver genes amongst patients of breast cancer, while mutations in *TP53* (Freq_Bailey *et al.*_: 0.92; Freq_Sanchez-Vega *et al.*_: 0.95; Fig. 4c) amongst patients of ovarian cancer were far more common than in any other gene, in alignment with previous studies.^23^^,^^58^ *TP53* mutations are highly associated with aneuploidy, which often involves substantial genomic deletions.^40^

The aforementioned driver prioritization methods predominantly focused on point mutations and focal events. We additionally compared our driver requirement estimates to the reconstructed evolutionary histories of 198 breast and 113 ovarian tumours from the ICGC-TCGA Pan Cancer Analysis of Whole Genomes (PCAWG).^33^ Gerstung et al.^33^ quantified the relative timing of 28 and 26 recurrent somatic events (*i.e.,* those occurring in >5% of samples) in breast and ovarian cancer, respectively. By determining the likelihoods of relative ordering between pairs of somatic mutations, they identified mutational events that preferentially occur early or late during tumour evolution. Comparing the relative timing of these somatic mutations to their recurrence rate, deletions were the most recurrent with a subset occurring preferentially early during tumour evolution.

Considering somatic mutations in the earliest quartile, 4/7 and 5/7 were deletions in breast and ovarian cancer, respectively (Fig. 4d and e). Deletions of chromosomes 13q (location of *RB1* and *BRCA2*) and 17 (location of *BRCA1*) were highly recurrent, early events in both cancer types. In ovarian cancer, 4q and 22q deletions were early events that each affected over 80% of cases. The deletion of 16q occurs early and affects more than 60% of patients of breast cancer. In comparison, while SNVs and indels in *PIK3CA* and *CBFB* were predicted to be amongst the earliest occurring mutations in breast cancer, they were observed in only 24.4% and 6.1% of the samples, respectively (Fig. 4d). They may be drivers that are only required and frequently observed for specific subtypes of breast tumours (*i.e., PIK3CA* mutation in luminal breast tumours). SNVs and indels in *TP53* were amongst the most recurrent and earliest occurring mutations in both breast and ovarian cancer (recurrence_breast_ = 47.5%; recurrence_ovarian_ = 92.0%; Fig. 4d and e). *TP53* mutations are associated with increased aneuploidy and are likely facilitating the deletion phenotype. The orthogonal mutation timing data support deletions as a critical rate-limiting mutation process in breast and ovarian cancer initiation.

Finally, we sought to identify genomic overlaps between early deletions identified through the evolution-timing analysis and driver deletions prioritized by Sanchez-Vega et al.^32^ (Supplementary Table S4). We note that the Sanchez-Vega et al. strictly considered events that were statistically recurrent, correlated with mRNA abundance, and showed a GISTIC value of −2 indicating deep (possibly homozygous) deletions, and thus may have an elevated false-negative rate. In breast cancer, the most recurrent, early deletion is chromosome 17p, in which *TP53* is located. In ovarian cancer, the predominant chromosome 17 deletion may additionally be associated with the prioritized *NF1* (located on 17q) deletion found in 7.9% of cases. *RB1*, whose prioritized deletion is found in 4.1% of breast tumours and 9.0% of ovarian tumours, is located on 13q—detected as another early, recurrent deletion by evolutionary analyses in both cancer types. Prioritized *PTEN* deletion affects 5.1% of breast tumours and intersects with the early 10q23.31 deletion. Some recurrent chromosome-arm deletions do not overlap with prioritized deletions of gene-level drivers and may affect other genes or trigger systematic effects. Overall, these analyses highlight selected chromosomal deletions (*i.e.,* 17 and 13q) and their intersections with cancer driver genes in breast and ovarian tumourigenesis. Thus, multiple independent approaches converge on a subset of deletions that may trigger tumourigenesis in breast and ovarian cancer. Implicating deletions as the rate-limiting trigger suggests somatic point mutations in cancer genes observed in normal tissue may be irrelevant in tumour initiation.^3^^,^^4^ This has important consequences for early detection of breast and ovarian cancers.

### Evaluation of rate-limiting genomic alterations at a cellular level

**Fig. 5:**
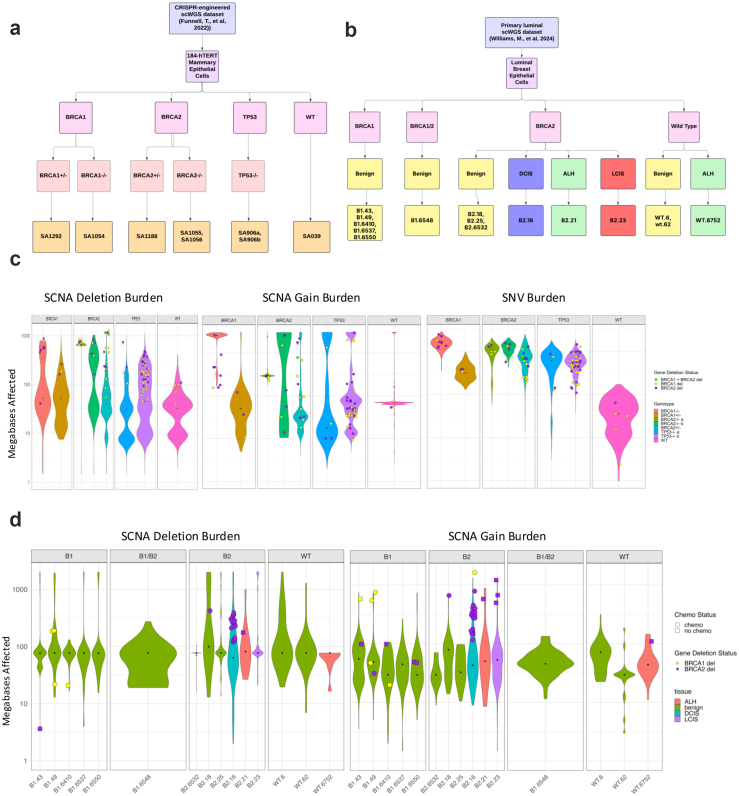
**Cell-level comparison of potential rate-limiting mutational events in CRISPR-engineered mammary epithelial cells and germline *BRCA1/BRCA2* luminal epithelial tissue samples**. (a) Summary flowchart of single-cell datasets and samples used in the analyses. The left panel displays data provided by Funnell et al. (2022), comprising scWGS-analyzed 184-hTERT mammary epithelial cells with CRISPR-engineered *BRCA1*, *BRCA2*, and *TP53* genotypes. (b) The right panel shows data from Williams et al. (2024), which includes SCNA profiles of benign and precancerous luminal breast epithelium. (c) Cell-level comparison of SCNA deletions, SCNA gain, and SNV burdens in CRISPR-engineered *BRCA1*, *BRCA2*, *TP53*, and wild-type cells from the scWGS dataset. (d) Cell-level comparison of SCNA deletions and gains in WT cells, as well as *BRCA1/BRCA2* luminal epithelial cells. In (c and d), the *BRCA1/BRCA2/TP53* genotype status of each sample were labeled on top fo each facet. Within each sample, cells with *BRCA1/BRCA2* somatic deletions were shown in color dots whereas cells without the deletions were summarized as a violin distribution colored by the sample's genotype (c) or tissue type (d).

To complement large patient cohort analyses, we examined the rates of SNVs, SCNA deletions, and SCNA gains using single-cell datasets of breast and ovarian cancer/pre-cancer cells (Fig. 5a and b). First, we analyzed a recently generated single-cell whole genome-sequencing (scWGS, DLP+) dataset^49^ of several CRISPR-engineered 184-hTERT L9 cell lines: SA1054 (*BRCA1*^*−/−*^), SA1055 (*BRCA2*^*−/−b*^), SA1056 (*BRCA2*^−/−a^), SA1292 (*BRCA1*^*+/−*)^, SA1188 (*BRCA2*^*+/−*^), SA906a (*TP53*^*−/−a*^), SA906b (*TP53*^*−/−b*^), and SA039 (WT), each with at least 377 cells (Supplementary Table S5). All cells were harvested between passages 20–59 and verified for their genotypes. The study provided QCed SNV calls derived using MutationSeq/Strelka and SCNAs quantified by SIGNALS,^49^ enabling comparative analyses of SNV and SCNA burdens in cells engineered with different *BRCA1*, *BRCA2*, and *TP53* genotypes (Fig. 5a, Methods).

For SNV burdens, all CRISPR-engineered cells exhibited significantly higher loads compared to WT cells (Wilcoxon Rank Sum test, FDR <0.01; Fig. 5c). *BRCA1*^−/−^ cells exhibited a significant increase in SNVs with a median fold change of 43×, *BRCA2*^−/−a^ cells at 30×, and *BRCA2*^−/−b^ cells at 35×. Heterozygous *BRCA1*^+/−^ cells showed 9×, *BRCA2*^+/−^ at 21×, whereas *TP53*^−/−a^ cells and *TP53*^−/−b^ are at 16–17×. For the burden of SCNA gains, *BRCA1*^−/−^ cells exhibited a significant increase (37× compared to WT), *BRCA2*^−/−a^ cells at 4×, and *BRCA2*^−/−a^ cells at 36×. However, SCNA was less or equivalent to WT in heterozygous *BRCA1*^+/−^ and *BRCA2*^+/−^ cells, as well as in *TP53*^−/−a^ and *TP53*^−/−b^ cells. For the burden of SCNA deletions, *BRCA1*^−/−^ cells showed a significant increase (2× compared to WT cells), *BRCA2*^−/−a^ cells at 20×, and *BRCA2*^−/−b^ cells at 4×. Heterozygous *BRCA1*^+/−^ and *BRCA2*^+/−^ cells exhibited a significant but lesser increase in SCNA deletions at 1.6× and 1.3× of WT (FDR <0.05), respectively. On the other hand, *TP53*^−/−a^ cells and *TP53*^−/−b^ cells showed 0.96× and 1.4× SCNA deletions of the WT. Overall, *BRCA1*/2-engineered cells showed increased SNV burdens similar to *TP53*^−/−^ cells, but uniquely exhibited increased burdens of SCNA gains (in only homozygous cells) and SCNA deletions (in both heterozygous and homozygous cells) compared to WT cells.

We next tested whether these rate differences are affected by somatic deletions of *BRCA1/*2 that likely will lead to LOH. In this analysis, we compared cells within the same genotype of CRISPR-engineered cells to identify significant differences (FDR<0.05). Relative to *BRCA*2^+/−^ cells without the somatic *BRCA2* deletion, *BRCA2*^+/−^ cells with somatic *BRCA2* deletion exhibited a 6.9× increase in SCNA deletions and a 7.2× increase in SCNA gains, while SNV burden remained unchanged (1.1×). In *BRCA2*^−/−b^ cells, somatic *BRCA2* deletion was associated with an 8.3× increase in deletions but non-significant changes in SCNA gain or SNV rates. There were insufficient *BRCA1* cells with somatic *BRCA1* deletions. Overall, these findings demonstrate a strong correlation between elevated SCNA deletion burden and somatic deletion/potential LOH of *BRCA1/2* in genetically-engineered *BRCA1/2* cells.

We next analyzed triple-negative breast cancer (TNBC) tissue samples analyzed in the same scWGS study, which included cells from 4 FBI (enriched in fold-back inversions), 2 HRD-Dup (enriched in small tandem duplications), and 1 TD (enriched in large tandem duplications) TNBC samples (Supplementary Fig. S9a), and reported the significant differences between germline *BRCA1/2* carriers and non-carriers (Wilcoxon Rank Sum test, FDR <0.05). Cells from the *BRCA1* TNBC sample had significantly more SCNA deletions compared to cells from the 4 FBI TNBCs (median fold change 4.5×) and the 1 TD TNBC (2.3×). On the other hand, the *BRCA1* cells had fewer SCNA gains compared to the FBI group (0.3×) and the TD subgroup (0.4×). For SNVs, the g*BRCA1* TNBC cells had similar or less SNV burdens compared to the FBI and the TD groups (Supplementary Fig. S9b). The High-Grade Serous Ovarian Cancer (HGSC) dataset in the same scWGS study contained cells from 8 FBI samples, 6 HRD-Dup samples, and 2 TD samples (Supplementary Fig. S9a). Overall, the HRD-Dup group showed less SCNA amplifications and similar SNVs to FBI and TD samples, while showing the highest burden of deletions, albeit the *BRCA1* sample did not show a higher deletion rate compared to other HRD-Dup samples in this HGSC dataset (Supplementary Fig. S9c).

We also analyzed a single-cell RNA-Seq (scRNA-Seq) dataset^51^^,^^59^ that includes *BRCA1* carrier (N = 4) *vs.* non-carrier (N = 4) TNBC tumour samples, as well normal breast tissue samples from preneoplastic *BRCA1*^*+/−*^ carriers (N = 4) and premenopausal non-carriers (N = 8) (Supplementary Fig. S10a) (Supplementary Table S5), reporting significant associations (FDR <0.05). One normal premenopausal epithelial breast tissue sample in the same dataset (N_1105_epi) was used as a reference for InferCNV^52^ to obtain somatic SCNAs from scRNA-Seq data (Methods). Compared to other TNBCs, *BRCA1* TNBC cells exhibited, on median, 9× SCNA deletions and 12× SCNA gains (Supplementary Fig. S10). SCNA landscapes at the cellular levels were consistent within the four *BRCA1* TNBC samples, whereas one non-carrier TNBC sample (TN_0135) was an outlier with increased SCNA (Supplementary Fig. S11). There may also exist minor differences in SCNA burdens between *BRCA1* carriers and noncarriers in normal tissue samples, albeit less pronounced: *BRCA1* preneoplastic cells showed 1.4× SCNA gains and 1.2× SCNA deletions (not significant) to non-carrier normal samples (Supplementary Fig. S12a and b). We also compared tumour and normal samples within the same genotype to assess genomic changes throughout tumourigenesis. In *BRCA1* carriers, TNBC cells showed 8× SCNA deletions (Supplementary Fig. S10c) and 4× SCNA gains compared to preneoplastic cells. In comparison, non-carrier TNBCs had 1.4× SCNA deletions and fewer SCNA gains (0.5×) when compared to non-carrier premenopausal cells (Supplementary Fig. S12a and b).

Finally, to delineate whether the observed rate differences may occur early in tumourigenesis, we analyzed recent scWGS (DLP+) dataset^50^ comprising luminal breast epithelial cells, including benign tissue samples from *BRCA1* (B1-43, B1-49, B1-6410, B1-6537,B1-6550), *BRCA2* (B2-18, B2-25, B2-6532), and wild-type individuals (WT-6, WT-62), as well as precancerous lesion samples, including ductal carcinoma in situ (DCIS: B2-18), lobular carcinoma in situ (LCIS: B2-23), and two atypical lobular hyperplasia (ALH: B2-21, WT-6752). One additional benign sample (B1-6548) harbored both *BRCA1* and *BRCA2* (Fig. 5b, Methods) (Supplementary Table S5). However, in this dataset, we did not observe any significant rate differences between *BRCA1* or *BRCA2 vs.* WT in SCNA deletions or gains within benign sample or ALH sample groups.

We also examined the potential SCNA burden effect of somatic deletions that could lead to LOH in the cells of these pre-cancer samples.^50^ Within luminal epithelial cells, somatic *BRCA1* deletion was detected in 0.73% of *BRCA1* cells (insufficient for comparative analyses) and somatic *BRCA2* deletion in 5.1% of *BRCA2* cells (Fig. 5c, Methods). We compared the SCNA burden in *BRCA2* cells with a somatic *BRCA2* deletion to those without the deletion to identify significant events (FDR <0.05). In the DCIS sample B2-16, cells with somatic *BRCA2* deletion exhibited an 8.6× increase in SCNA deletion, and a 6.0× increase in SCNA gains. Overall, these data confirm at a cellular level that *BRCA1/2* TNBCs and engineered cells typically showed higher SCNA burdens than non-carrier or WT cells, and SCNA deletions are the most consistent type of mutation process that show higher rates in *BRCA1/2* cells.

## Discussion

Cancer is caused by the successive accumulation of aberrations that deregulate normal cellular processes and trigger tumourigenesis.^1^^,^^2^ However, despite multiple efforts, the driving mutational process and the number of aberrations required to initiate a diagnosable tumour remain largely unknown.^6^, ^7^, ^8^, ^9^, ^10^, ^11^, ^12^, ^13^, ^14^ Identifying the trigger of tumourigenesis would help delineating malignant from non-malignant somatic evolution and provide key information for accurate risk stratification of pre-malignant lesions. Integrating established theoretical and empirical methods, we compared mutational processes in germline *BRCA1/2*-heritable breast and ovarian tumours to their sporadic counterparts to identify fundamental rate-limiting genetic alterations required for tumourigenesis. We focused on breast and ovarian cancer to avoid confounding with well-known sex-biases in cancer mutations and to ensure sufficient numbers of carriers for modeling (n > 10).^60^^,^^61^ We identified deletions as the rate-limiting mutational process initiating tumourigenesis in breast and ovarian cancer. Despite current protocols largely focused on point mutations, these data prioritize structural variants and copy number alterations in early detection strategies, such as liquid biopsies.

Although prioritized drivers based on sequencing studies include many classes of alterations (*e.g.*, *PIK3CA* hotspots, *ERBB2* SCNA gains),^10^^,^^32^ these may not represent rate-limiting steps required to become a diagnosable breast or ovarian tumour. Similarly, profiling somatic mutations in non-malignant tissues have demonstrated a high prevalence of mutations in cancer genes, *e.g.*, *NOTCH*, despite the absence of cancer.^3^^,^^4^ While these mutations may confer a fitness advantage, they are unlikely to be the rate-limiting events in early tumourigenesis. Reconstructing the history of breast and ovarian tumours predicted multiple highly recurrent deletions as early events many of which were arm-level deletions that intersected with tumour suppressor genes, including *RB1* on 13q as well as *NF1* and *TP53* on chromosome 17. Interestingly, a recent study^62^ has identified cancer/non-cancer breast tissues identified a key clonal event to be derivative chromosome der (1;16), which includes the entire long arm of chromosome 1 (1q) and a truncated short arm of chromosome 16 (16p) acquired during early puberty to late adolescence in patients of breast cancer.^62^ *TP53* mutational status is also known to be correlated with aneuploidy.^40^ Beyond *TP53/RB1*, arm-level losses have coherent effects: 17p co-deletes *POLR2A*^63^ and *HIC1*,^64^ lowering RNA Pol II dosage and derepressing SIRT1; 13q haploinsufficiency affecting *BRCA2* and *LATS2*^65^ may reduce homologous repair capacity and Hippo restraint; 16q loss affects *CDH1* and *CTCF*^66^ (cell adhesion, chromatin insulation) and often disrupts *WWOX/FANCA* (Wnt/DDR defects). These convergent impacts—on chromatin/transcription, DNA repair/replication stress, and adhesion/signaling—help explain selection for broad deletions and nominate liabilities (*e.g.*, POLR2A-directed agents, PARP/DDR strategies, YAP/TEAD inhibition) for functional testing. However, reconstructing the history of breast and ovarian tumours predicted more early deletions than our theoretical framework predicted. Because timing estimates are rank-based, we cannot estimate the time-frame between the acquisition of two drivers. Despite both being labeled early events, one deletion event could occur long before a second early event. Further, it is possible not all deletions confer a fitness advantage.

*BRCA1* or *BRCA2* loss of function leads to HRD and higher dependence on more error prone repair pathways, such as non-homologous end joining (NHEJ) and microhomology mediated-end joining (MMEJ). HRD tumours are enriched in <50 kbp genomic deletions characteristic of NHEJ or MMEJ repair.^13^^,^^56^ Specifically, recent ICGC PCAWG studies identified an indel signature ID6 that correlated with the SBS3—often attributed to defective HRD; ID6 is characterized predominantly by deletions of ≥5 base pairs and with overlapping microhomology at the boundaries,^67^ suggesting a potential deletion type that may occur more frequently when tumours develop HRD deficiency.

This study has limitations. The mechanisms by which germline *BRCA1* and *BRCA2* deficiency promotes tumourigenesis are likely myriad. The penetrance of *BRCA1* and *BRCA2* variants can be modulated by the genomic environment^68^^,^ and their role in tumourigenesis may be compounded by the tumour microenvironment,^69^ both of which are not fully captured in our framework. Our framework requires simplified assumptions, including that both carriers and non-carriers require a similar number of driver alterations, each genetic variation type is modelled in isolation, and similar fitness effects of each base pair of deletion/gain/mutations. Our recent survey of the genomic architecture of 1828 breast tumors^70^ suggests the mutational patterns in carriers are not distinct from sporadic tumors, particularly TNBC tumors. Our breast cancer modeling is likely enriched for TNBC tumours because we controlled for *TP53* mutations and subtypes. Thus, in breast cancer, deletions may be a more dominant rate limiting event in TNBC tumours. There is a smaller subset of ER+ and HER2+ tumors harboring complex amplifications that may result from a separate evolutionary path. We focus on determining the lower-bound on the rate-limiting number of exonic driver alterations required. Future work may also consider genomic rearrangements, non-coding, transcriptional or post-transcriptional changes that could contribute to tumourigenesis. Our quantitative framework models different somatic alteration types distinctly to estimate the minimum number of required drivers for each class of mutation. HRD in carriers drives an increased burden of multiple alteration types, with varying impact during tumor evolution. Explicitly modeling the dependencies, timing, and interactions among these different mutation types is a key future research direction, but will require much higher sample sizes to accurately fit models. Our primary rate-limiting estimation framework, based on population-level incidence and mutation rates, does not directly infer clonality for each specific alteration. However, the mutation timing analysis by Gerstung et al.,^33^ which we leveraged (Fig. 4d and e), explicitly infers the relative timing of recurrent somatic events, distinguishing between those that occur early (likely clonal) and late (potentially subclonal) during tumor evolution. The deletions we highlight as early and recurrent events in Fig. 4d and e, such as deletions of chromosomes 17p and 13q, are indeed inferred to be clonal or near-clonal events in a substantial fraction of tumors. Furthermore, the single-cell analyses (Fig. 5) provide direct evidence of the presence of SCNA deletions in a high proportion of cells within both engineered models and pre-cancerous patient samples, consistent with these being clonal or early clonal events. Lastly, germline *BRCA1* and *BRCA2* variants show high ancestry-specificity and different ancestral groups are differentially impacted by breast and ovarian cancers^34^, ^35^, ^36^; our analyses herein are based on cohorts with predominantly Europeans, and these findings need to be carefully examined in diverse populations.

Germline *BRCA1* and *BRCA2* carriers are recommended to begin screening for breast cancer starting at age 25 and discussions of risk-reducing mastectomy or salpingo-oophorectomy starting at age 35.^28^ There is an increased prevalence of pre-malignant lesions, *e.g.*, ductal carcinoma in-situ (DCIS) and tubual intraepithelial carcinoma (TIC), in *BRCA1* and *BRCA2* carriers.^29^, ^30^, ^31^ Our finding that large deletions represent rate-limiting events suggests practical assays in both tissue and plasma. In FFPE tumour tissue, low-pass whole-genome sequencing (lpWGS; ∼0.1–1 ×) robustly profiles arm-level and broad focal losses with modest DNA input, and can be analyzed with allele-specific CNA callers; this approach performs well on FFPE and is cheaper than deep WGS while outperforming capture-based CNV from exomes/panels for broad events.^71^ In liquid biopsy, ultra-/shallow-WGS of cfDNA with ichorCNA or similar methods jointly estimates tumor fraction and detects chromosome/arm-level losses,^72^ supporting feasibility for monitoring and risk stratification in high-risk cohorts such as BRCA1/2 carriers. For cost-effectiveness, ultra-/shallow-WGS leverages standard library prep and minimal sequencing to capture genome-wide deletion signals in a single assay, making it an attractive adjunct to MRI/mammography and to targeted mutation panels in high-risk surveillance.

Altogether, this work demonstrates the efficacy of integrating theoretical models and observational sequencing studies to identify mutational requirements triggering tumourigenesis. As sequencing cohorts expand, such methods can be applied to other cancer types, including prostate and pancreatic cancers. Thus, these methods serve to answer a fundamental question in cancer biology across cancers providing critical insights to prospectively detect, predict and prevent tumour evolution.

## Contributors

Accessed, verified, and processed the underlying data: K.E.H., M.B., Y.G., D.F., M.W., S.H., K.H.

Performed statistical and bioinformatics analyses: K.E.H., M.B., Y.G., J.G.C., D.F., G.L.G., H.H.

Wrote the first draft of the manuscript: K.E.H., M.B., Y.G., K.H.

Edited the manuscript: K.E.H., P.V.L., P.C.B., K.H.

Initiated the project: K.E.H., K.H.

Supervised research: P.C.B., K.H.

All authors read and approved the final manuscript.

## Data sharing statement

### Data availability

Processed mutation calls were downloaded from https://gdc.cancer.gov/about-data/publications/pancanatlas. To generate SCNA frequency maps, data was downloaded from the GDC repository (https://dcc.icgc.org/releases).

### Code availability

The codes to reproduce the results can be found in the following repositories:

(1) Original analyses: https://github.com/khoulahan/brca-driver-estimation
(2) Analyses matched for PAM50 subtypes, pathologic stages, and somatic TP53 driver status: https://github.com/Huang-lab/brca-driver-estimation/
(3) Single-cell analyses: https://github.com/mahadabihie/scWGS-BRCA-Analysis/

## Declaration of interests

Gonzalo Lopez holds stocks and is employed by Bristol Myers Squibb. Kathleen Houlahan's work was supported by a CIHR Vanier Fellowship. Paul C. Boutros's work was supported by NIH. PCB was part of the scientific advisory board at Sage Bionetworks, Biosymetrics Inc., and Intersect Diagnostics Inc. Other authors declare no competing financial interests.

## Appendix A Supplementary data

Supplementary Table S5

Caption for Supplementary Material
