# Supplementary material for: Quantifying rate-limiting genetic variation in breast and ovarian tumourigenesis
Source: eBioMedicine. 2026 Feb 21;125:106181. doi: 10.1016/j.ebiom.2026.106181 (PMC12945530; doi:10.1016/j.ebiom.2026.106181)

Supplementary Figure 1

a)

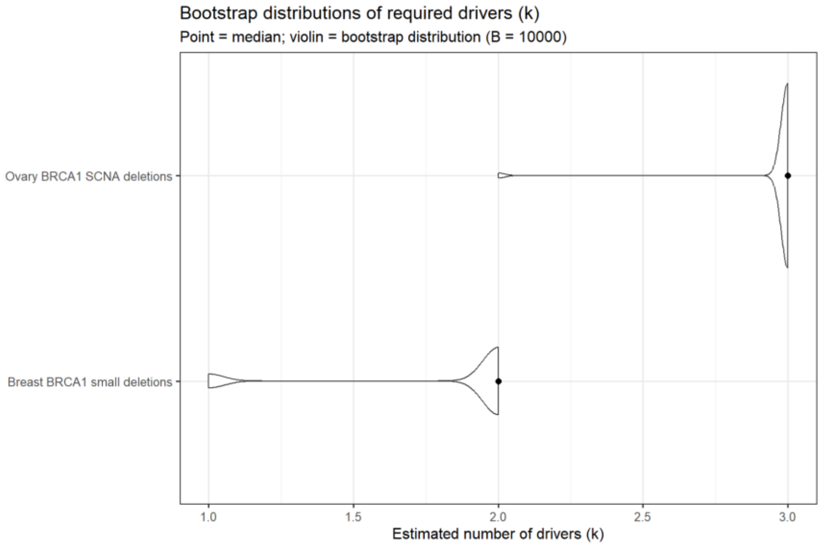

b)

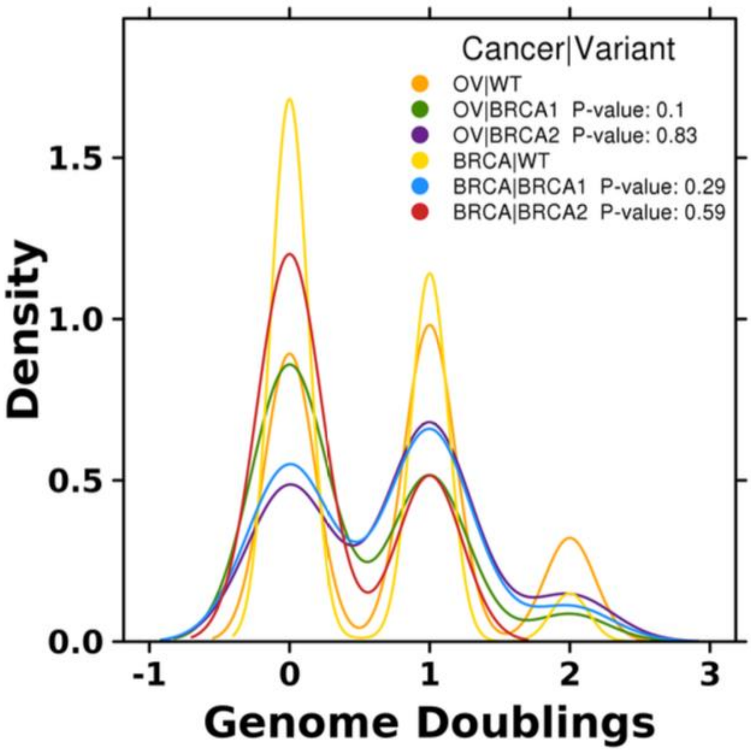

c)

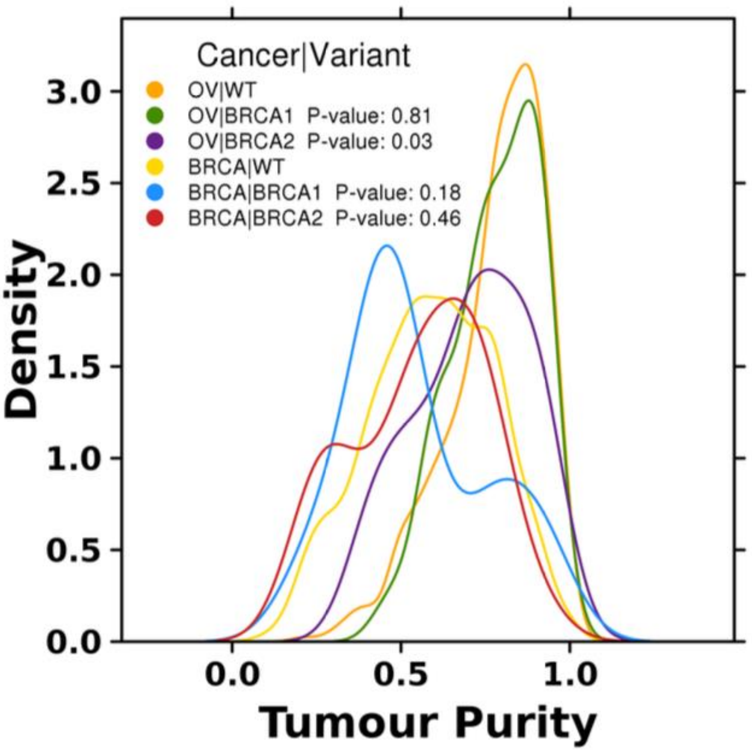

Supplementary Figure 2

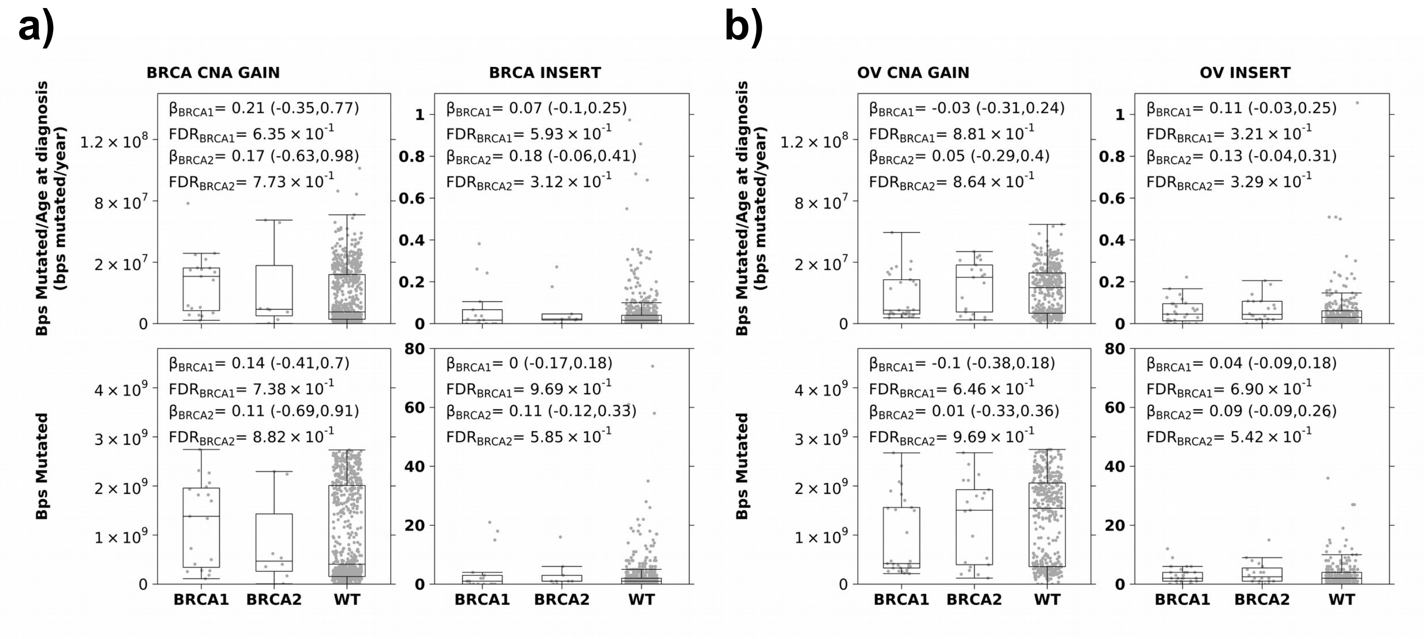

Supplementary Figure 3

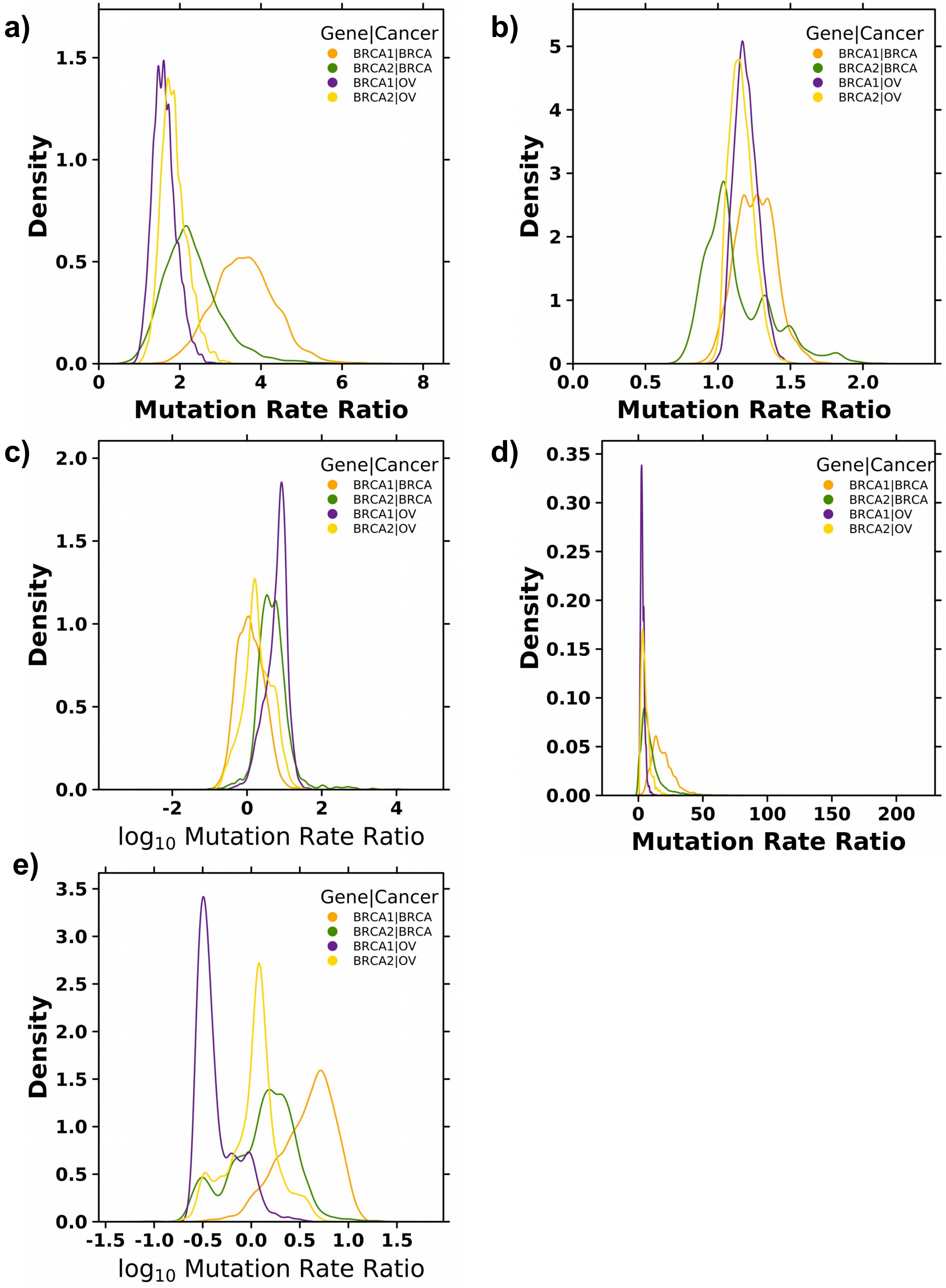

Supplementary Figure 4

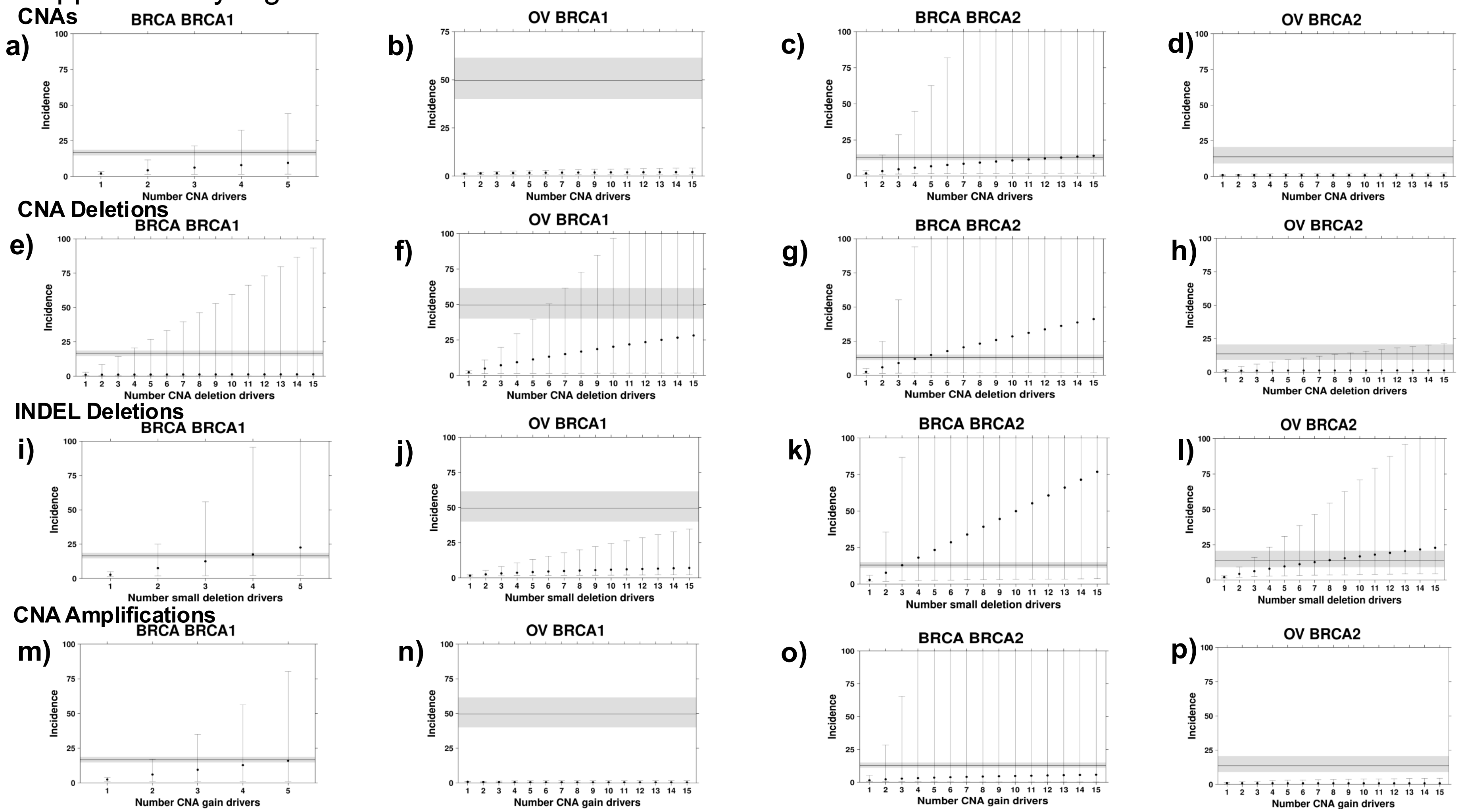

Supplementary Figure 5 (BRCA1/2 + LOH vs. Non-Carriers)

SNVs

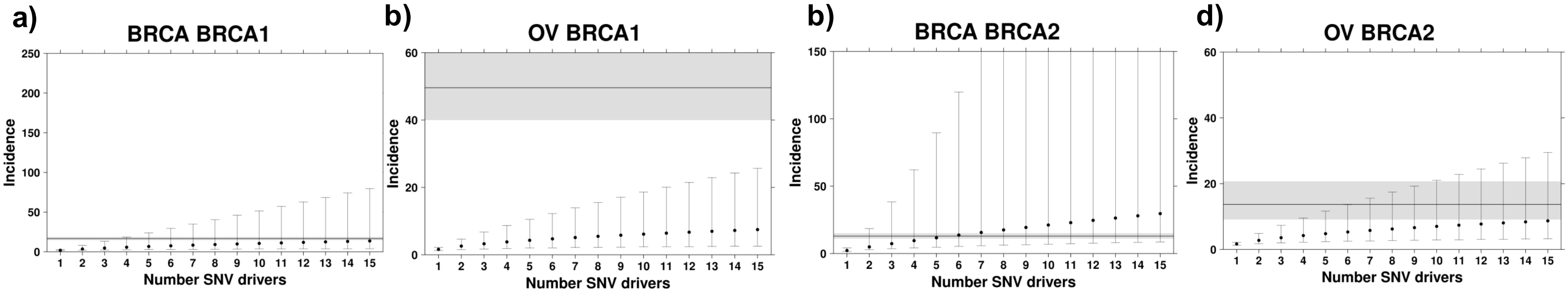

CNAs

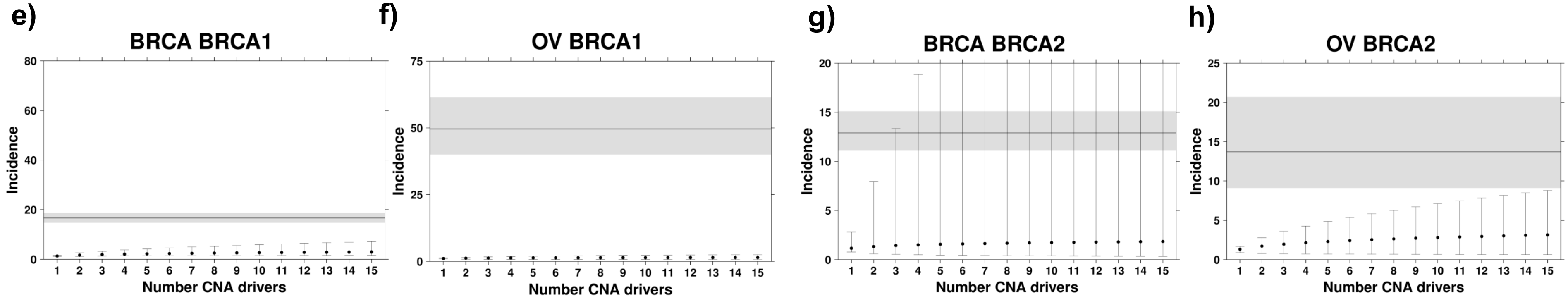

Supplementary Figure 6 (BRCA1/2 + LOH vs. Non-Carriers)

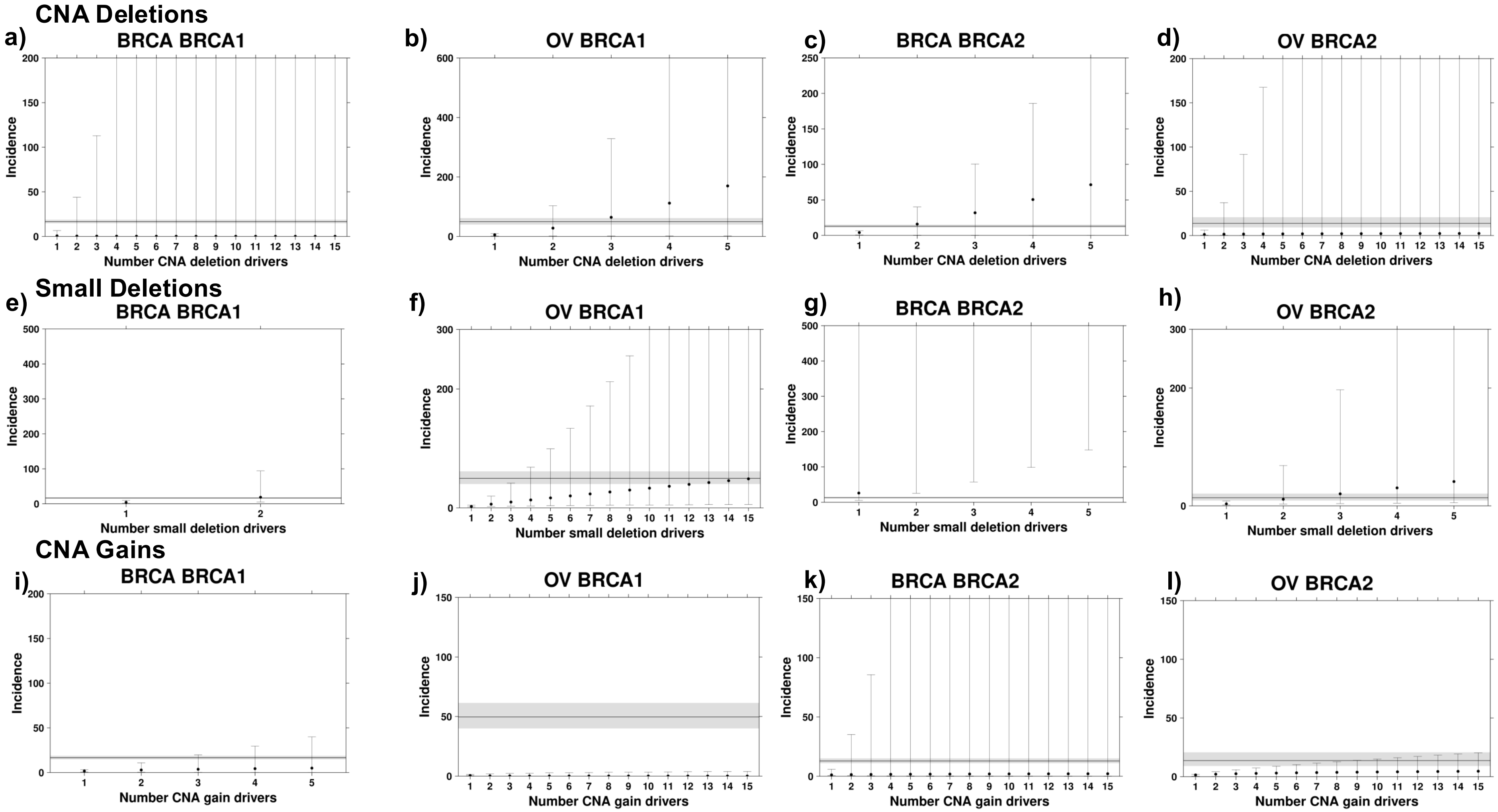

Supplementary Figure 7

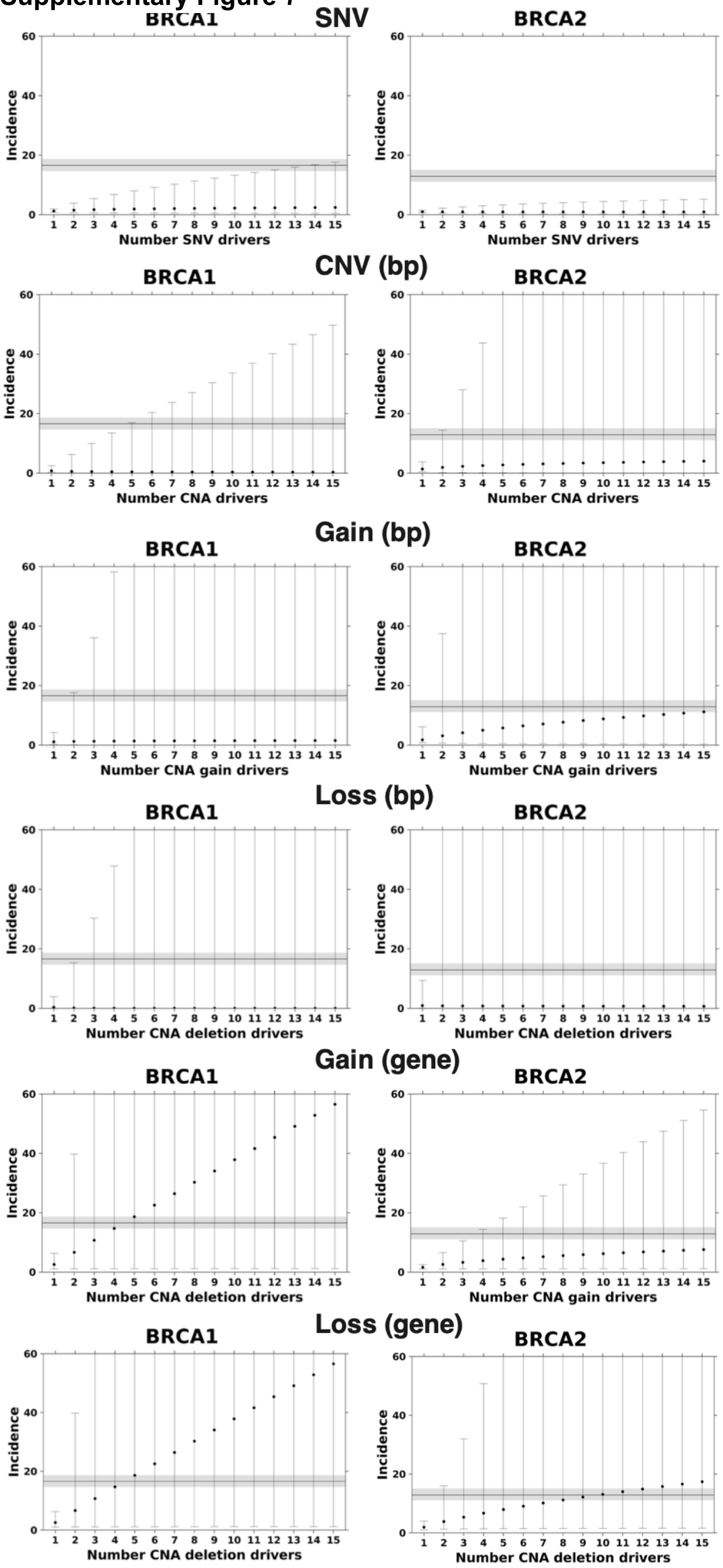

Supplementary Figure 8

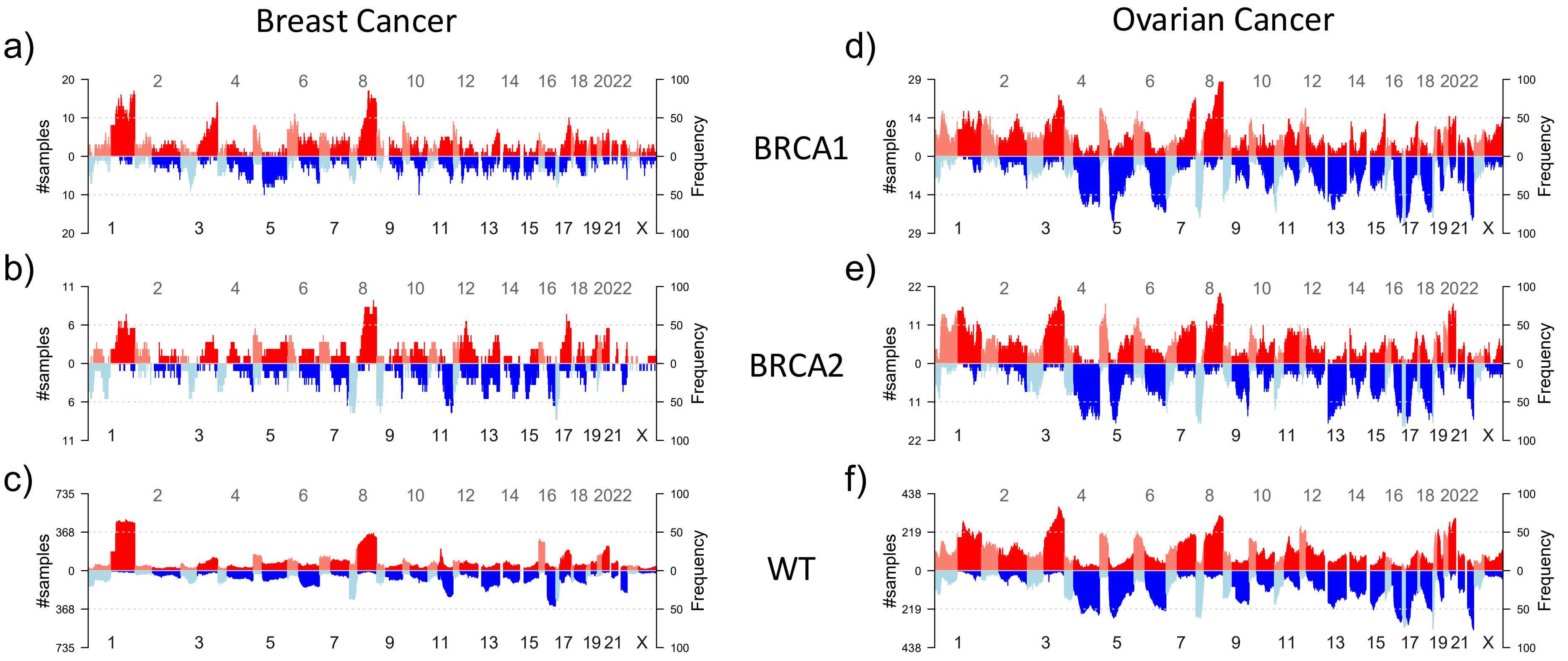

Supplementary figure 9

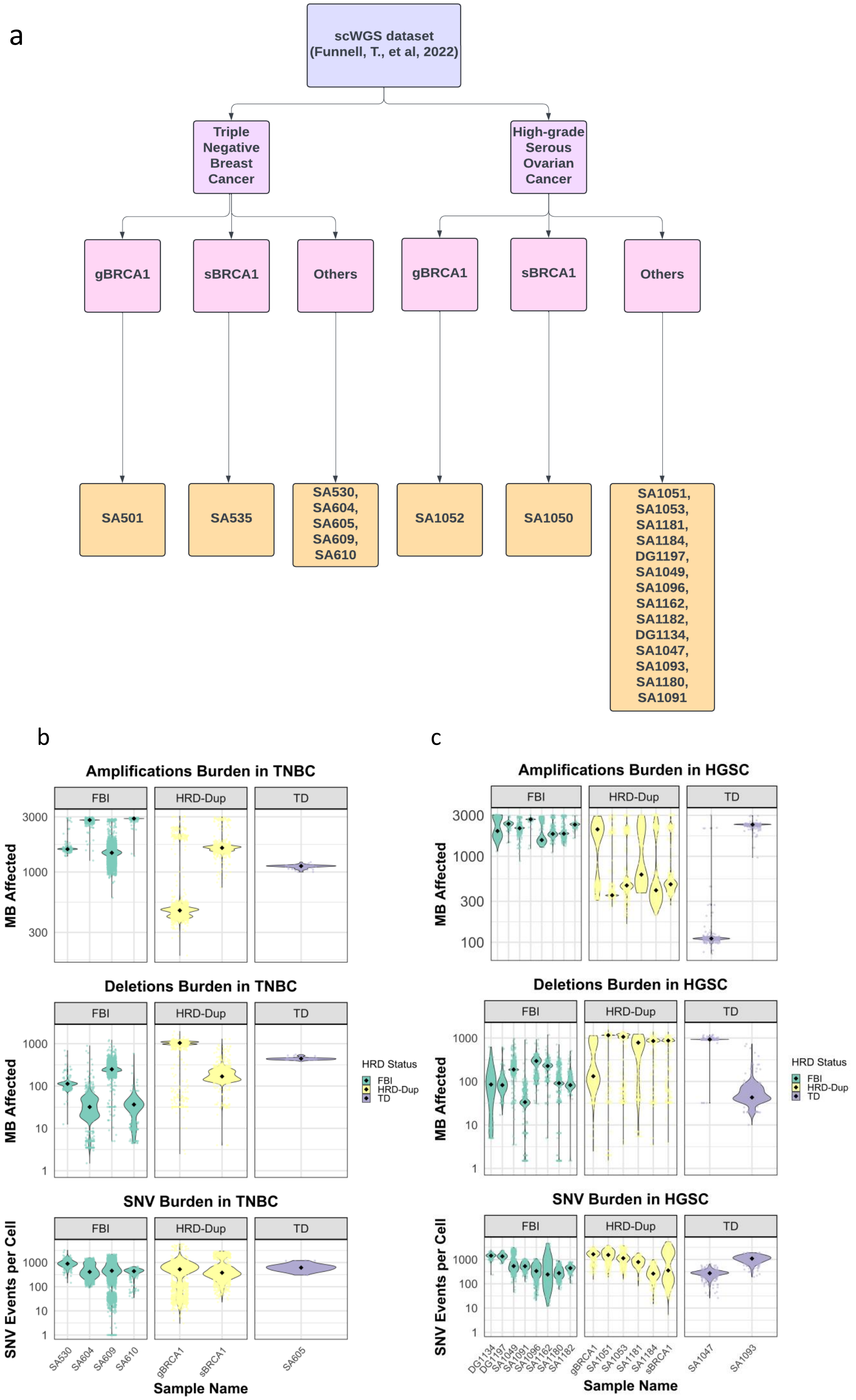

Supplementary figure 10

a)

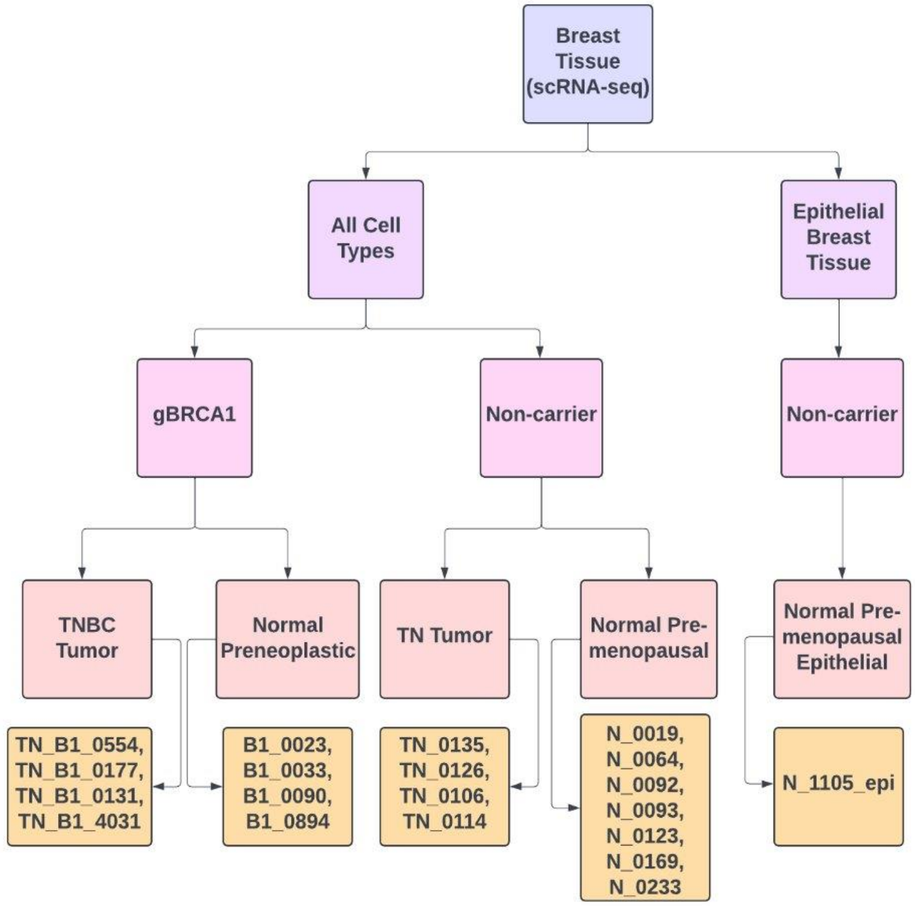

b)

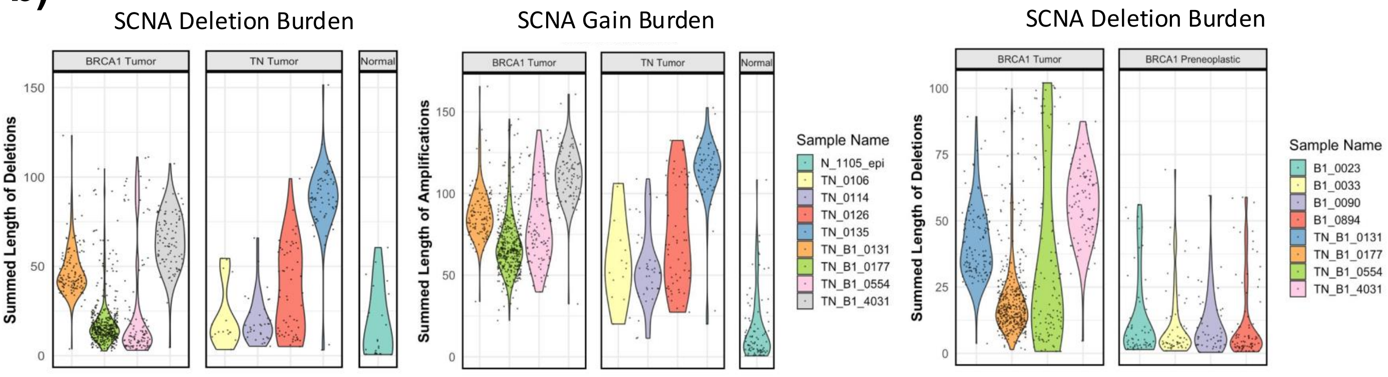

Supplementary figure 11

a) inferCNV heatmap of non-BRCA1 TNBC Samples (Normal Epithelial Reference)

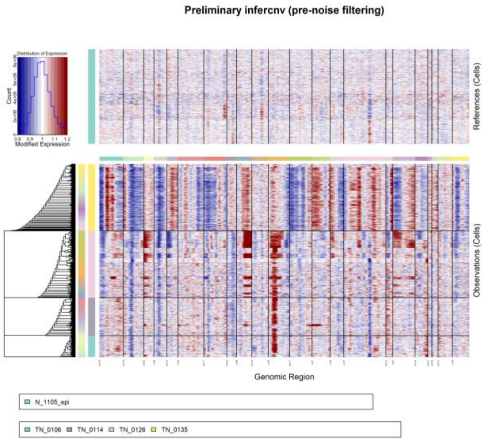

b) inferCNV heatmap of BRCA1 TNBC Samples (Normal Epithelial Reference)

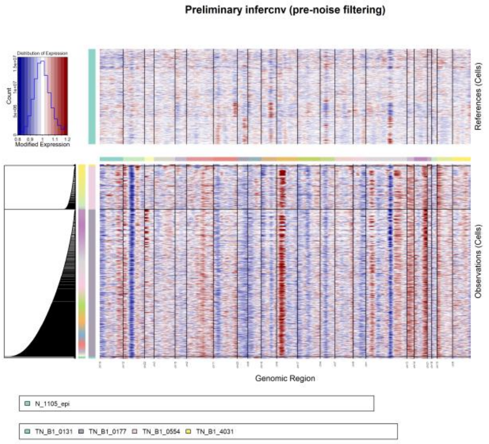

c) inferCNV heatmap of non-BRCA1 Premenopausal Normal Breast Tissue (Normal Epithelial Reference)

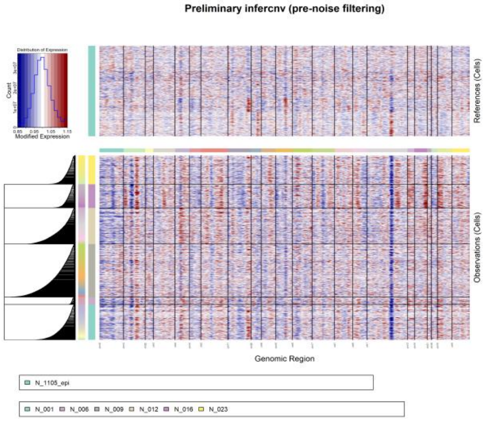

d) inferCNV heatmap of BRCA1+/- Preneoplastic Normal Breast Tissue (Normal Epithelial Reference)

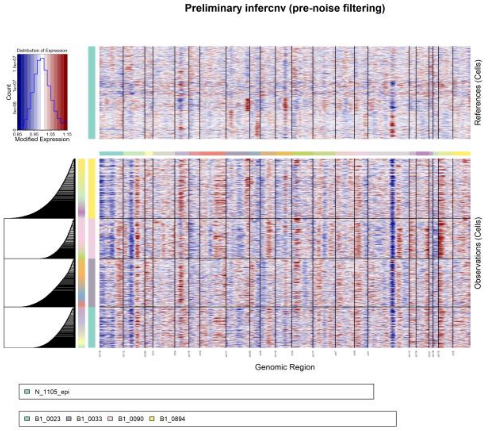

Supplementary figure 12

a) CND Length of BRCA1+/- Preneoplastic Normal Breast Tissue & non-BRCA1 Premenopausal Normal Breast Tissue

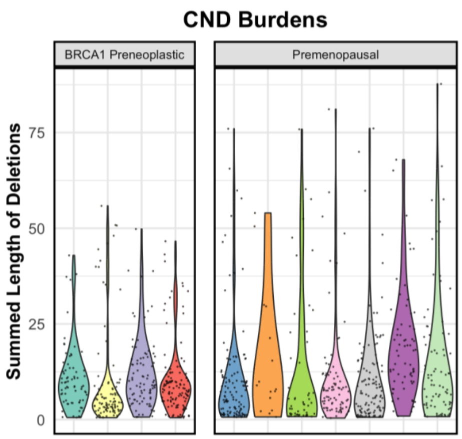

b) CNA Length of BRCA1+/- Preneoplastic Normal Breast Tissue & non-BRCA1 Premenopausal Normal Breast Tissue

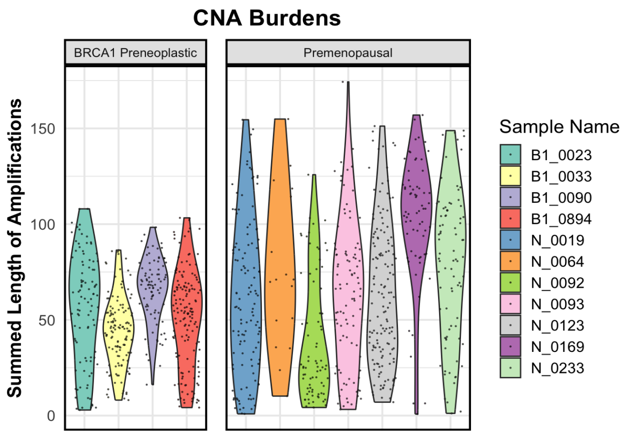

c) CND Length of non-BRCA1 TNBCs & non-BRCA1 Premenopausal Normal Breast Tissue

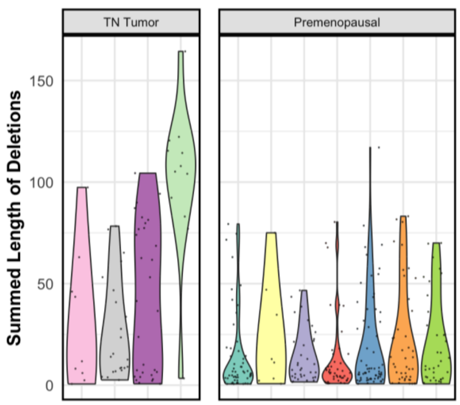

d) CNA Length of non-BRCA1 TNBCs & non-BRCA1 Premenopausal Normal Breast Tissue

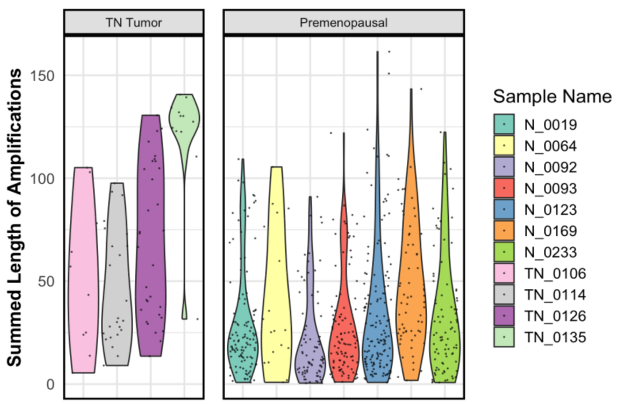

Supplement: Supplementary Figs. S1–S12 [file mmc1.pdf]
