# Supplementary material for: Quantifying rate-limiting genetic variation in breast and ovarian tumourigenesis
Source: eBioMedicine. 2026 Feb 21;125:106181. doi: 10.1016/j.ebiom.2026.106181 (PMC12945530; doi:10.1016/j.ebiom.2026.106181)
Supplement: Supplementary Table S2 [file mmc3.pdf]

Supplementary Table 2 (Matching on Stage, PAM50 and TP53 Status)

BRCA BRCA1

Group	# Carrier	% Carrier	Avaiable WT	% WT Sample	Adjusted WT
Basal|Stage II|TP53	8	0.42	55	0.42	23
Basal|Stage II|WT	2	0.11	7	0.14	1
LumA|Stage II|WT	2	0.11	173	0.1	18
LumB|Stage III|TP53	2	0.11	19	0.11	2
Basal|NA|TP53	1	0.05	1	0	0
Basal|Stage III|TP53	1	0.05	7	0	0
Basal|Stage I|TP53	1	0.05	13	0.08	1
LumA|Stage I|TP53	1	0.05	9	0	0
LumA|Stage I|WT	1	0.05	74	0.05	4

BRCA BRCA2

Group	# Carrier	% Carrier	Available WT	% WT Sample	Adjusted WT
Basal|Stage II|WT	2	0.15	7	0.14	1
LumA|Stage III|WT	2	0.15	75	0.16	12
LumA|Stage II|WT	2	0.15	173	0.16	27
LumA|Stage X|WT	2	0.15	6	0.17	1
LumA|Stage II|TP53	1	0.08	19	0.05	1
LumA|Stage I|TP53	1	0.08	9	0.11	1
LumB|Stage III|WT	1	0.08	25	0.08	2
LumB|Stage II|TP53	1	0.08	20	0.1	2
LumB|Stage II|WT	1	0.08	49	0.08	4

OV BRCA1

Group	# Carrier	% Carrier	Available WT	% WT Sample	Adjusted WT
Stage III|TP53	26	0.81	192	0.81	156
Stage IV|TP53	3	0.09	39	0.1	4
Stage III|WT	1	0.03	20	0.05	1
Stage II|TP53	1	0.03	12	0	0
Stage IV|WT	1	0.03	4	0	0
NA|TP53	0	0	2	0	0
Stage II|WT	0	0	1	0	0
Stage I|TP53	0	0	9	0	0
Stage I|WT	0	0	1	0	0

OV BRCA2

Group	# Carrier	% Carrier	Available WT	% WT Sample	Adjusted WT
Stage III|TP53	16	0.73	192	0.73	140
Stage IV|TP53	4	0.18	39	0.18	7
NA|TP53	1	0.05	2	0	0
Stage I|TP53	1	0.05	9	0	0
Stage III|WT	0	0	20	0	0
Stage II|TP53	0	0	12	0	0
Stage II|WT	0	0	1	0	0
Stage IV|WT	0	0	4	0	0
Stage I|WT	0	0	1	0	0

- **Group**: Breast Cancer Subtype | Cancer Stage| TP53 Mutation Status
- **# Carrier**: Number of *BRCA1*/*BRCA2* carriers
- **% Carrier**: Percentage in this group
- **Available WT**: Number of available Wild Type patients
- **% WT Sample**: Proportion of WT to sample from
- **Adjusted WT**: Number of sampled WT patients
