# Supplementary material for: Quantifying rate-limiting genetic variation in breast and ovarian tumourigenesis
Source: eBioMedicine. 2026 Feb 21;125:106181. doi: 10.1016/j.ebiom.2026.106181 (PMC12945530; doi:10.1016/j.ebiom.2026.106181)
Supplement: Caption for Supplementary Material [file mmc7.docx]

**Caption for supplementary material**

**Supplementary Figure Legends:**

**Supplementary Figure 1: Robustness of the Study, Including power analyses and consistent tumour purity and ploidy across subgroups**

**a)** Bootstrapped distributions of required drivers under modelled scenarios. **b)** The number of samples that experienced genome doubling was consistent across subgroups. The density plot shows the distribution of sample-wise genome doubling for each subgroup. **c)** Tumour purity was not significantly different between cancer subgroups with the exception of *BRCA2* ovarian cancer samples were had significantly lower tumour purity than wildtype. Density plot shows the distribution of sample-wise tumour purity for each subgroup.

**Supplementary Figure 2: Increased mutation rates in germline *BRCA1* and *BRCA2* carriers**

Breast **(A)** and ovarian **(B)** tumours in *BRCA1* and *BRCA2* carriers show increased mutation counts and rates, defined as count/age, compared to non-carrier (WT) individuals. Boxplots represent median, 0.25 and 0.75 quantiles with whiskers are 1.5x interquartile range. P-values from the Kruskal-Wallis test. GAIN: gains; INSERT: insertions

**Supplementary Figure 3: Mutation burden ratio distributions**

Distributions of mutation burden ratios between carrier and non-carrier over all of the 10,000 bootstrap iterations for SNVs **(a)**, SCNAs **(b)**, SCNA deletions **(c)**, small deletions **(d)** and gains **(e)**.

**Supplementary Figure 4: Estimating incidence from SCNA segments**

Incidence estimates based on SCNA segment counts **(a-d)**, SCNA deletion segment counts **(e-h),** INDEL deletion counts **(i-l)** and SCNA gains segment counts **(m-p)**. All segment counts are normalized by age at diagnosis. Like in Figure 2, the incidence ratios for carriers were benchmarked against non-carrier controls matched for tumour subtype, stage, and somatic *TP53* status.

**Supplementary Figure 5**: Incidence estimates comparing *BRCA1* and *BRCA2* + LOH carriers vs. non-carriers. Estimated incidence rates based on SNVs **(a-d),** SCNAs **(e-h)**.

**Supplementary Figure 6**: Incidence estimates comparing *BRCA1* and *BRCA2* + LOH carriers vs. non-carriers. Estimated incidence rates based on SCNA deletions **(a-d),** small deletions **(e-h)** and gains **(i-l)**.

**Supplementary Figure 7: Estimating breast cancer incidence in the METABRIC cohort**

Estimated incidence rates based on SNV and SCNA (gain and losses) in the METABRIC breast cancer cohort.

**Supplementary Figure 8: Copy number frequency map of *BRCA1*, *BRCA2*, and non-carrier tumours**

Genome-wide representation of SCNAs indicating the number of samples (left axis) and frequencies (right axis) with copy number loss (light blue/blue) and gain (salmon/red). The lighter colors represent short chromosome arm (p) and intense color represents long (q) arms. **a-c)** Breast cancers carriers for *BRCA1* **(a)**, *BRCA2* **(b)** and non-carriers **(c)**. **d-f)** Ovarian cancer samples carriers for *BRCA1* **(d)**, *BRCA2* **(e)** and non-carriers **(f)**.

**Supplementary Figure 9: Cell-level comparison of rate-limiting mutation process in triple negative breast cancer and high-grade serous ovarian cancers.**

(a) Summary flowchart of scWGS datasets from Funnell, T. et al. (2022). TNBC and HGSC cells with germline *BRCA1*, somatic *BRCA1* and non-*BRCA1/2* carrier genotypes are outlined here. (b) Cell-level comparison of deletion, SCNA gain and SNV burden in gBRCA1, sBRCA1 and other genotypes of both TNBC and (c) HGSC cells in the scWGS dataset. Each dot represents either the SCNA burden of an individual cell in terms of metabases affected by SCNA or the number of SNV events per cell (b-c).

**Supplementary Figure 10: Cell-level comparison of rate-limiting mutational processes in germline BRCA1/2 versus non-carrier tumour samples.**

(a) Summary flowchart of single-cell datasets and samples analyzed from Pal et al. (2021), including scRNA-Seq data from germline BRCA1 carriers and non-carriers, spanning triple-negative breast cancer (TNBC) tumour tissues and premenopausal/pre-neoplastic normal breast tissue. (b) Cell-level comparison of SCNA deletion and gain burdens in TNBCs from *BRCA1* carriers versus non-carriers (left, middle), and *BRCA1* TNBCs versus pre-neoplastic normal breast tissue (right), based on SCNA inference using inferCNV from scRNA-Seq data. Each point provides the genomic alteration burden of a given cell in the sample. For SCNA gains/deletions, the SCNA burdens were calculated by the summed length of the megabase affected. The dot in each violin represents the median cell in a given sample.

**Supplementary Figure 11: inferCNV-generated heatmaps of SCNA in both TNBC and normal samples of germline *BRCA1* carriers and non-carriers based on scRNA-Seq data.** Heat maps of SCNA landscapes, including that for non-carrier TNBCs (a), *BRCA1* TNBCs (b), non-carrier premenopausal normal samples (c) and *BRCA1* preneoplastic normal tissue (d). SCNA gains for each chromosome region are highlighted red, while deletions are highlighted blue. The normal reference sample is at the top of each plot, while each sample analyzed is below (a-d).

**Supplementary Figure 12: Cell-level comparison of rate-limiting mutational process in non-carrier TNBC, non-carrier premenopausal and germline *BRCA1* preneoplastic samples based on inferCNV analyses of scRNA-Seq data.** Cell-level comparison of *BRCA1* preneoplastic and non-carrier premenopausal cells in terms of CND (a) and SCNA (b) burdens. CND (c) and SCNA (d) burdens compared across non-carrier tumour and normal samples. Individual dots of the violin plots represent the summed length of megabases deleted or amplified for a given cell (a-d).

**Supplementary Tables Legends:**

**Supplementary Table 1: Standardize incidence rate (SIR) of breast and ovarian cancer in *BRCA1* and *BRCA2* carriers**

**Supplementary Table 2:**

We performed a matching of non-carrier breast and ovarian cancer samples to g*BRCA1/*g*BRCA2* samples using a weighted random sampling procedure, implemented via custom scripts in R. For breast cancer, non-carrier tumours were matched based on the distribution of PAM50 subtypes (Luminal A, Luminal B, *HER2*-enriched, Basal-like, Normal-like), pathologic stages (I-IV) and *TP53* somatic mutation status, to match the sample size of *BRCA1* or *BRCA2* carriers. For ovarian cancer, matching was performed based on clinical stage distribution and *TP53* somatic mutation status.

**Supplementary Table 3:** Confidence intervals and p-values for the Kolmogorov-Smirnov test statistic (D).

**Supplementary Table 4: Early SCNA deletions identified through evolution-timing analysis and driver prioritization methods**

Overlap between early deletions identified through the evolution-timing analysis and driver deletions prioritized by Sanchez-Vega *et al.*(32) AVG_CNA_PER_PT = average SCNA per patient; AMP = SCNA gain; DEL = deletion

**Supplementary Table 5: Single cell datasets and samples used for analyses**

The list of samples from the three studies, including (1) Funnell, T. et al. (2022) that provided scWGS data of genetically engineered 184-hTERT Mammary Epithelial Cells, TNBC, and HGSC samples of various genotypes; (2) Pal, B. et al. (2021) that provided scRNA-Seq-analyzed samples from germline BRCA1 carriers and non-carriers, including Triple Negative Breast Cancer (TNBC) tumour tissues and premenopausal/pre-neoplastic normal breast tissues. (3) Williams, M. et al. (2024) that provided scWGS samples from luminal breast epithelial cells derived from benign and precancerous tissues.
